# TDTAC: a generalized time-dependent torsion angle correlation framework for resolving directional coordination in large biomolecular assemblies

**DOI:** 10.3389/fmolb.2026.1821703

**Published:** 2026-06-03

**Authors:** Carolina Escobar Palacio, Tongye Shen, Chia-En A. Chang

**Affiliations:** 1 Department of Chemistry, University of California, Riverside, CA, United States; 2 Department of Biochemistry and Cellular and Molecular Biology, University of Tennessee, Knoxville, TN, United States

**Keywords:** allosteric communication, protein degradation, dynamic network, E3 ligase, molecular dynamics, torsion analysis, ubiquitination

## Abstract

Proteins function through coordinated, time-dependent conformational motions, yet conventional analyses often obscure how these dynamics propagate across complex assemblies. Here, we introduce a generalized Time-Dependent Torsion Angle Correlation (TDTAC) framework that incorporates explicit time lags between residues, enabling quantitative mapping of directional, sequential correlations. By analyzing dihedral rotations, TDTAC reveals conformational dynamics through a physically meaningful representation of residue-level fluctuations. We apply this framework to examine how a small-molecule degrader may induce time-lagged structural rearrangements that facilitate targeted protein degradation. We analyzed the PROteolysis TArgeting Chimera (PROTAC) dBET70, a bifunctional small molecule in complex with its target protein, bromodomain-containing protein 4 (BRD4^BD1^), and assembled within the full degradation complex Cullin-RING Ligase 4A (CRL4A) E3 ligase scaffold. The full assembly comprises nine components: dBET70, BRD4^BD1^, and the seven-protein degradation complex CRBN, DDB1, CUL4A, NEDD8, RBX1, E2, and Ub. TDTAC analysis reveals that motions originating at the DDB1–CUL4A region propagate along two dominant pathways: one extending through DDB1 toward the CRBN–BRD4 interface, and the second extending through CUL4A toward the RBX1–Ub interface. These coordinated, time-delayed rearrangements are associated with configurations that bring BRD4, E2, Ub, and the PROTAC into a ubiquitination-competent state. Both pathways exhibit similar lag-dependent propagation behavior, consistent with a network of time-delayed residue-residue coordination linking local torsional dynamics to distal catalytic interfaces. More broadly, TDTAC provides a generalizable framework for resolving dynamic information flow in large biomolecular assemblies.

## 1 Introduction

Proteins are inherently dynamic systems whose biological functions depend on conformational flexibility and time-dependent motions (Branden and Tooze, 1991; Kessel and Ben-Tal, 2018). Structural rearrangements underlie key processes such as ligand binding, catalysis, and allosteric regulation (Goodey and Benkovic, 2008; Wodak et al., 2019). Molecular dynamics (MD) simulations and enhanced sampling techniques provide time-resolved trajectories that reveal how collective motions shape biochemical activity (Dror et al., 2012). Yet, despite advances in long-timescale simulations, quantifying dynamic coordination between residues remains challenging (Loutchko and Flechsig, 2020; Nussinov et al., 2023). Traditional analyses, including root-mean-square metrics, quasi-harmonic analysis, and cross-correlation maps, primarily characterize time-averaged fluctuations or covariance relationships and lack the capacity to capture transient, time-lagged relationships that characterize how motions propagate between residues or domains (Dutta et al., 2017; Yu and Dalby, 2020). Consequently, the temporal relationships underlying allosteric signaling and long-range mechanical coordination often remain obscured.

**FIGURE 1:**
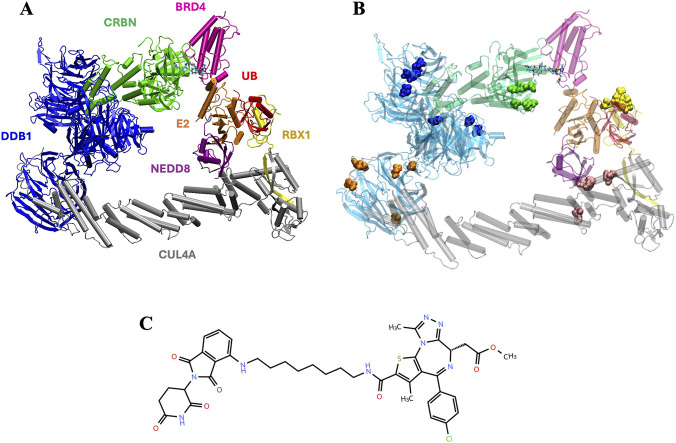
Structures of PROTAC-mediated degradation machinery complex. **(A)** Full degradation machinery complex is constructed with DDB1 (blue), CUL4A (gray), NEDD8 (purple), RBX1 (yellow), E2 enzyme (orange), Ub (red), CRBN (green), and BRD4^BD1^ (magenta). dBET70 is shown as licorice representation. **(B)** Clusters of residues found at each of the pivot regions within the complex. DDB1–CUL4A central region residues are shown in orange, DDB1–CRBN interface-proximal residues are shown in blue, and CUL4A C-terminal residues are shown in pink. Green and yellow residue clusters are shown for CRBN and RBX1, respectively. **(C)** Chemical structure of the dBET70 PROTAC.

Large protein assemblies undergo multi-domain conformational rearrangements essential for function yet difficult to quantify with existing analytical approaches (Goodey and Benkovic, 2008; Wodak et al., 2019; Dror et al., 2012; Johnson et al., 2018). In these systems, allosteric signaling, substrate positioning, and catalytic readiness emerge from coordinated motions of multiple domains or subunits, producing complex dynamical landscapes (Nussinov et al., 2023; Johnson et al., 2018; Tang and Chang, 2017; Chang and Gilson, 2003). A representative example is the PROteolysis TArgeting Chimera (PROTAC)–mediated protein degradation with CRL4A E3 ligase degradation machinery. PROTAC is a bifunctional small molecule that tethers a target protein, here BRD4^BD1^, to the E3 ligase receptor, Cereblon (CRBN) (Qi et al., 2021; Liu et al., 2022; Bricelj et al., 2021; Lee et al., 2022; Wu et al., 2024) (Figure 1). CRBN engages the CRL4A scaffold, composed of the CULLIN4A (CUL4A) scaffold, the adaptor DDB1, and the RING protein RBX1 which recruits the E2–Ubiquitin (Ub) conjugate (Wu et al., 2024) (Figure 1A). Together, this complex forms a modular platform that positions Ub and BRD4^BD1^ for ubiquitination and subsequent proteasomal degradation (Zhou et al., 2018). The invention of PROTACs has revolutionized chemical biology by enabling selective degradation of previously “undruggable” proteins rather than merely inhibiting their activity (Dale et al., 2021; Békés et al., 2022). In this case, the PROTAC-induced degradation of BRD4^BD1^ (an epigenetic protein that regulates transcription of key oncogenes) ensures sustained suppression of certain cancer-driving genes, offering a more effective therapeutic strategy (Lu et al., 2015). The PROTAC dBET70 (Figure 1C) is highly effective in degrading Bromodomain and Extra-Terminal domain (BET) proteins, exhibiting a DC_50/5h_ (half-maximal degradation concentration at 5 h) of ∼5 nM, substantially outperforming less potent analogs by several orders of magnitude (DC_50/5h_ of ∼50–500 nM) (Filippakopoulos et al., 2010; Winter et al., 2015). This dBET70-bound CRL4A complex undergoes coordinated conformational changes that position BRD4 for ubiquitination (Qin et al., 2018). Despite recent structural characterization by cryo-EM and simulation studies, the temporal coordination of these conformational changes, and how motions propagate across the complex, remains poorly understood.

The temporal relationships of conformational changes within proteins have been previously investigated using time-dependent correlation (TDC) analyses. Existing approaches applied canonical and time-lagged correlation methods to DNA (Briki and Genest, 1994; Genest, 1996), transmembrane helices (Gamier and Genest, 1996), and protein residues (Melvin et al., 2018; Chong and Ham, 2021), demonstrating that correlated motions can extend across large structures and exhibit heterogeneous communication timescales. Related frameworks include generalized correlation analyses (Lange and Grubmüller, 2006), information-theoretic measures such as transfer entropy (Schreiber, 2000), and causal inference approaches applied to biomolecular dynamics (Ghosh and Vishveshwara, 2007). Collectively, these methods have provided important insight into correlated and directed motions in proteins; however, they typically rely on instantaneous or equilibrium-based correlations, or assume long-timescale stationarity, which can obscure transient propagation events in large multi-component assemblies. For example, recent work has introduced a time-lagged correlation propagator to identify which correlations grow toward equilibrium and to assess sampling sufficiency in MD simulations (Melvin et al., 2018). Other studies have used dynamic correlations to show that DNA and proteins retain conformational memory, that residues communicate during protein folding, and that asymmetric coupling and long-range communication occur in systems such as calmodulin (Chong and Ham, 2021; Das et al., 2016). Analyses of time-dependent dihedral oscillations have also revealed residue-specific dynamic signatures often obscured in Cartesian-space analyses (Bastidas and Sevarac, 2024). Unlike these approaches, which focused on frequency-domain signatures or Cartesian-based single-protein correlations, the present framework constructs a TDC method using torsional angles as primary degrees of freedom on a multi-unit complex.

**FIGURE 2:**
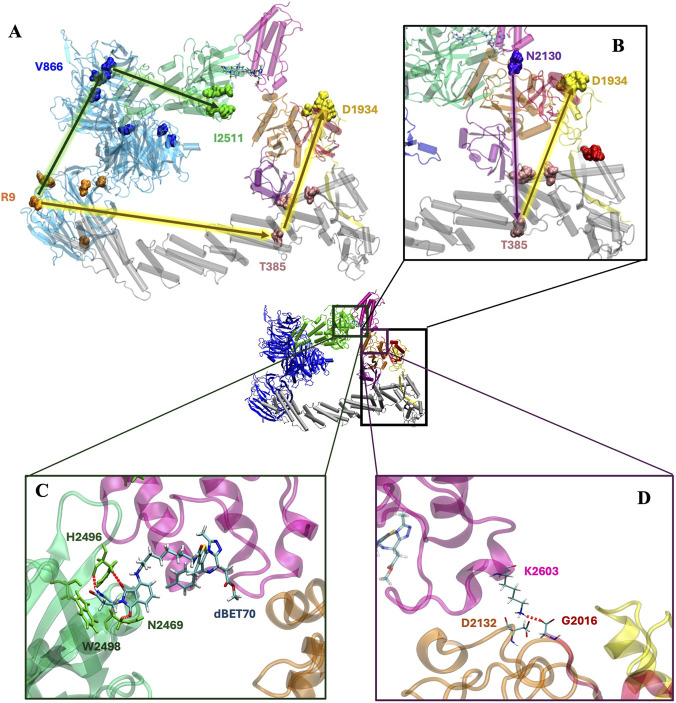
Propagation paths and stabilizing interactions within the dBET70-induced CRL4A E3 ligase complex. **(A)** Correlation propagation pathways in the substrate recognition module (green) and catalytic module (yellow). The pathways shown represent a single residue-level example selected from multiple propagation pathways observed across pivot regions, originating from Arg9 and proceeding through key residues to distal sites (e.g., Arg9 to Val866 to Ile2511 in the substrate recognition module, and Arg9 to Thr385 to Asp1934 in the catalytic module). **(B)** Feedback propagation within the catalytic module, highlighting connections from Asn2130 to Thr385 (purple) and Thr385 to Asp1934 (yellow). **(C)** Stabilizing interactions between CRBN (green) and dBET70 linker; hydrogen bonds are shown as red dashes. **(D)** Stabilizing interactions among E2 (orange), BRD4 (magenta), and Ub (red), including a interaction between Lys2603 and Gly2016 important for ubiquitination.

This study introduces a generalized TDTAC framework that extends the Pearson correlation coefficient by incorporating an explicit time lag parameter. Using torsional angles provides a physically meaningful representation of residue motions, avoiding artifacts such as bond stretching and linear displacements associated with Cartesian coordinates (Chang and Gilson, 2003; Chang et al., 2003). This formulation preserves intrinsic rotational dynamics and captures the directionality of correlated motions (Ai et al., 2010). This framework is applied to the PROTAC-induced dBET70 CRL4A complex, representing one of the first systematic time-lagged correlation analyses of this system. By incorporating time lags, TDTAC delineates how motions originating from the central DDB1-CUL4A region (Figures 1, 2A) propagate across interdomain pathways linking distant functional interfaces and structurally important regions.

Mapping these correlations over time reveals a structured sequence of conformational changes that are consistent with configurations associated with catalytic competence. Overall, this framework provides a generalizable and computationally efficient tool for probing allosteric signaling, conformational transitions, and dynamic information flow in biomolecular assemblies. Identification of two major propagation pathways within the PROTAC CRL4A E3 ligase complex (Figure 2A) demonstrates how this approach uncovers the temporal hierarchy of motions associated with functional conformational changes, offering insights that are directly relevant to understanding protein degradation mechanisms.

## 2 Methods

Detailed theoretical derivations, equations, algorithmic implementation, simulation protocols, and network analysis procedures are provided in the Supplementary Material. In summary, TDTAC was formulated by extending the classical Pearson correlation coefficient (Jenkins and Watts, 1968) to include an explicit time-lag parameter, enabling quantification of directional, lag-resolved propagation of correlated motions between residues while preserving normalization. For dihedral-based analysis, circular statistics were used to properly account for angular periodicity. Backbone φ torsion angles were transformed into sine and cosine representations to compute circular means and differences, and reconstructed as phase angles using the two-argument arctangent function (arctan2), ensuring that correlations reflect intrinsic rotational dynamics without boundary artifacts (Tang and Chang, 2017). MD trajectories of the dBET70-induced CRL4A E3 ligase complex were obtained from Wu et al. (2024). Simulations were performed using AMBER20 with the ff14SB force field (Maier et al., 2015) and GAFF2 (He et al., 2020) parameters for proteins and ligand, respectively, followed by 400 ns of production runs under explicit-solvent conditions. Two independent MD simulations were performed using different random initial velocity seeds to ensure robustness of the observed dynamical correlations. Backbone dihedral φ time series were extracted using T-Analyst (Ai et al., 2010), which computes torsions directly from Cartesian coordinates, and served as input for TDTAC calculations. For benchmarking, classical Pearson correlation and autocorrelation analyses were performed on identical dihedral time series extracted from the same trajectories, following established correlation frameworks for protein dynamics (Lange and Grubmüller, 2006; Ghosh and Vishveshwara, 2007). In the zero-lag limit, the TDTAC formulation reduces exactly to the Pearson correlation computed on the same variables, providing a direct baseline for comparison with lag-resolved results. Relative RMSD analyses, performed after alignment to selected scaffold anchor regions, were used to identify hinge-like flexion points and inter-domain coordination within the assembly. To characterize global dynamics, lag-dependent TDTAC values were stored as three-dimensional arrays $\left(L\times i\times i\right)$ , where each lag L defines a residue-residue correlation matrix $\left(i\times i\right)$ . Time lags from 0 to 75 ns were used in this study. Selected non-zero lag matrices were extracted to construct adjacency matrices using a defined correlation cutoff, enabling graph-theoretical analysis of clustering, centrality, and community structure. These dynamic networks were compared with static structure-based contact networks derived from the MD ensemble.

## 3 Results

### 3.1 Identification of pivot regions and analysis of coordinated motions using TDTAC

By aligning MD trajectories using selected protein regions, this structural bioinformatics approach enables effective analysis of region-specific structural dynamics. Here, we focus on the dynamics of the target protein BRD4 and the E2 enzyme, whose dBET70-induced motions play a pivotal role in successful ubiquitination. To quantify the motions of BRD4 and E2 relative to selected protein regions, the MD trajectory was aligned to three identified structural regions (herein referred to as the DDB1–CUL4A central region, the DDB1–CRBN interface-proximal region, and the CUL4A C-terminal region (Supplementary Figure SI1), and RMSD was calculated with respect to the initial frame of the trajectory. As illustrated in Supplementary Figure SI1, relative RMSD analyses revealed two major transitions in BRD4 and E2 motions. In particular, RMSD comparisons between BRD4 and the DDB1-CUL4A central region showed a rapid reorganization before 100 ns and a slower rearrangement near 300 ns (Supplementary Figure SI1A). Similar transitions were seen in both BRD4 and E2 RMSD motions relative to the CUL4A C-terminal region and the DDB1–CRBN interface-proximal region, with transitions seen before 100 ns and near 300 ns, respectively (Supplementary Figure SI1B, C). To assess whether these transitions correspond to catalytically relevant states, structural distances between two residue pairs critical for isopeptide bond formation (Wu et al., 2024) (BRD4 Lys2603–Ub Gly2016 and E2 Asp2132–BRD4 Lys2603) were monitored throughout the trajectory. Both pairs approached catalytically competent distances (<3 Å and <5 Å, respectively) at around 300 ns (Figure 2D), suggesting that the second observed RMSD transition likely corresponds to a ubiquitination-ready conformation.

To further characterize how these structural rearrangements are organized at the complex level, we examined the corresponding protein structure network and compared it with the dihedral dynamic network derived from TDTAC analysis. For both the structure and dynamic networks, clustering coefficients (triad-based C3) and centrality measures, including degree centrality (DC), betweenness centrality (BC), closeness centrality (CC), and eigenvector centrality (EC), were computed. The results for the structure network exhibit a classical negative correlation between clustering coefficient and DC (defined as the number of linked nodes normalized by maximal connectivity), a hallmark of hierarchical and modular biological networks (Ravasz et al., 2002) (Supplementary Figure SI2A). Although the structure network does not strictly display scale-free behavior and its degree distribution remains relatively constrained (Albert et al., 1999), several prominent structural hubs are evident, characterized by high DC but low C3. The torsional dynamic network displays markedly different wiring properties. At link densities above 10%, the dynamic network becomes increasingly noisy; however, at link densities of 1%, a positive correlation between C3 and DC emerges (Supplementary Figure SI2B). While negative C3-DC correlations are typical of hierarchical biological networks, consistent with prior dynamic network formulations of protein communication (Sethi et al., 2009), the observed positive correlation indicates a topology in which highly connected residues are also embedded within tightly interconnected neighborhoods. This behavior suggests cooperative clusters of dynamically synchronized residues rather than hub-mediated hierarchical organization. Notably, the peak value of DC in the dynamic network is observed in proteins DDB1 and CUL4A, indicating that these regions form the largest number of strong time-lagged dynamic connections with other proteins of the complex (Supplementary Figure SI2B). These findings suggest that the dynamic network highlights functionally coordinated residues that are not necessarily evident from static contact topology alone, reinforcing the notion that structural connectivity and dynamic communication encode distinct but complementary aspects of protein organization.

To assess the robustness of these observations, all TDTAC and network analyses were performed on two independent MD trajectories initialized with different random initial velocities. Although the exact residue-level participation in individual propagation pathways differs between trajectories, both simulations consistently recover the same functional clustering of residues across five major structural regions: the DDB1–CUL4A central pivot region, the DDB1–CRBN interface-proximal region, the CRBN–BRD4 interface, the CUL4A C-terminal proximal region, and the RBX1-Ub interface. Notably, the second trajectory additionally reveals participation of some BRD4 interface residues in late-stage propagation events, which are less pronounced in the first trajectory. Despite these differences in residue-level detail, both trajectories exhibit similar lag-dependent correlation patterns, temporal ordering, and propagation timescales, indicating that the observed TDTAC features are robust to stochastic variation in initial velocity conditions. All analyses presented in this section primarily correspond to the first trajectory, while corresponding results from the second trajectory are provided in Supplementary Figures SI3, SI5, SI8, SI10.

Guided by the RMSD analysis and the topological features identified in the network framework, we next examined residue-level torsional dynamics within the three selected pivot regions (Supplementary Figure SI1). Backbone φ dihedral rotations were analyzed over the 400 ns MD trajectory. Notably, dihedral fluctuations observed early in the simulation (within the first 100 ns) correspond to an initial rearrangement of the DDB1–CUL4A central region, while slower fluctuations late in the simulation (observed at 250–300 ns) reflect downstream reorientations of distal regions involving residues within CRBN–BRD4 interface and RBX1–Ub interface (Supplementary Figure SI4, SI5). The TDTAC framework was applied to all backbone φ dihedral angles in the dBET70-bound CRL4A E3 ligase assembly, using time lags from 0 to 75 ns. Analyses of the resulting time-lagged correlation data focused mostly on residues within the identified pivot regions (Figure 1B). As shown in Supplementary Figure SI3A, residues Arg9, Val1090, Val1185, Asn1295, and Ser1316, located in the DDB1–CUL4A central region, underwent some of the earliest motions observed in the full complex (happening within the first 100 ns). TDTAC analysis of these residues confirmed that the DDB1–CUL4A central region is associated with early-time fluctuations that precede correlated, time-delayed responses throughout the E3 ligase assembly. Mapping the temporal evolution of these correlations starting at the DDB1–CUL4A region revealed two dominant propagation pathways: one extending through the substrate-recognition module (DDB1–CRBN–dBET70–BRD4) and another through the catalytic module (CUL4A–RBX1–E2–Ub) (Figure 2A). The delayed behavior of the correlations exhibits a sequential pattern suggesting lag-dependent coordination between regions. These results indicate a hierarchical yet synchronized dynamic correlation pattern, wherein pivot motions at the DDB1–CUL4A central region propagate through both modules to coordinate domain rearrangements associated with catalytically favorable configurations. Overall, the relative RMSD and network analyses guided the identification of key pivot regions and residues, which informed the selection of protein regions for focused TDTAC analysis. This approach suggests how local pivot flexibility translates into global coordinated motions across both substrate-recognition and catalytic modules, offering a quantitative framework for analyzing how conformational information propagates through the dBET70-induced degradation machinery complex.

### 3.2 Propagation of motion through the substrate-recognition module: from DDB1 to CRBN

**FIGURE 3:**
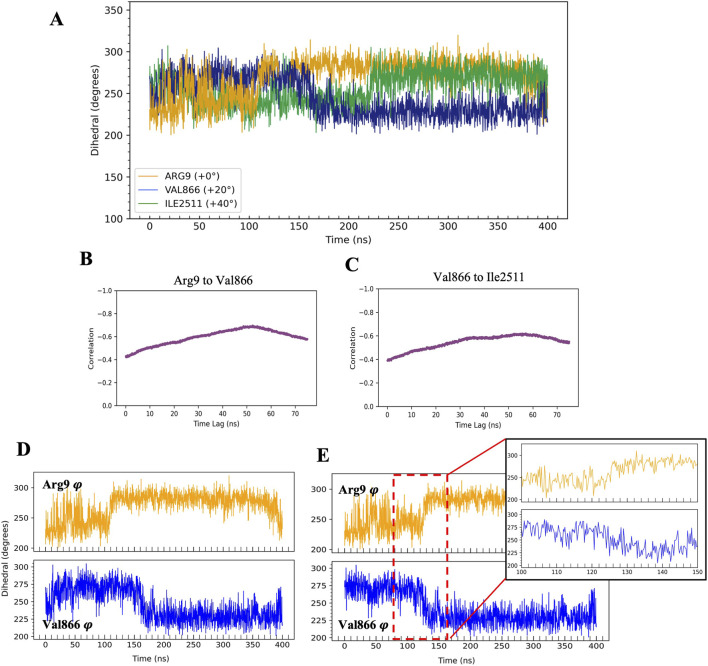
Time-dependent torsional angle correlations (TDTAC) within the substrate-recognition module. **(A)** φ dihedral time series for Arg9, Val866, and Ile2511. **(B)** TDTAC plot for propagation from Arg9 to Val866. **(C)** TDTAC plot for propagation from Val866 to Ile2511. **(D)** Arg9 (orange) and Val866 (blue) dihedrals at zero lag time (L = 0). **(E)** Val866 dihedral shifted by 50 ns lag time (L = 50). Red dashed lines indicate alignment of dihedral fluctuations.

Within the substrate-recognition module, our TDTAC analysis revealed that the earliest coordinated fluctuations, originating at the DDB1–CUL4A central region, exhibited strong time-delayed correlations with a group of residues located in the distal DDB1–CRBN interface-proximal region (Figures 1B, 2A). Using ±0.4 as a cutoff for the computed correlation coefficient, we identified residues Glu860, Val866, Ile879, Ala896, Ile899, Asp1572, Gln1637, and Glu1649, which exhibit synchronized fluctuations centered around 150 ns (Supplementary Figure SI4B). These relationships are evident in TDTAC plots displaying strong time-lagged correlations between the DDB1–CUL4A central region and DDB1–CRBN interface-proximal residues (Figure 3 and SI7A–D). For example, the φ dihedral of Arg9 in the DDB1–CUL4A central region and the φ dihedral of Val866 near the DDB1–CRBN interface displayed an initial correlation below 0.5 during the first ∼5 ns of lag time, followed by a sharp increase, reaching a correlation coefficient >0.6 after 30 ns, and peaking at 0.7 after 50 ns (Figure 3B). When the Val866 dihedral data is shifted left by ∼50 ns relative to its original position at 0 ns lag, its fluctuations align closely with those of Arg9, indicative of a time-lagged relationship between these motions (Figures 3D,E). This in-phase alignment exemplifies the TDTAC framework’s ability to resolve sequential relationships between conformational changes across distant regions of the protein complex. Consistent TDC patterns were observed between other DDB1–CUL4A central residues (active around 100 ns) and DDB1–CRBN interface-proximal residues (active near 150 ns), establishing a clear temporal relationship consistent with motion propagation (Supplementary Figure SI4A–D). The delayed fluctuations within the DDB1–CRBN region are associated with a subsequent wave of correlated motions in CRBN residues positioned proximal to the interface with BRD4.

By approximately 250 ns of simulation time, residues within CRBN–BRD4 interface–namely, Cys2484, Leu2486, Asn2487, Ile2511, and Cys2512 (Supplementary Figure SI4C)– underwent distinct dihedral reorientations, facilitating formation of stabilizing noncovalent interactions with the dBET70 linker. These dihedral shifts correlated strongly with earlier DDB1–CRBN interface-proximal residue motions. For instance, the TDTAC plot for the Val866 φ dihedral (near the DDB1–CRBN interface) and the Ile2511 φ dihedral (near CRBN–dBET70 interface) reveals an increasing correlation pattern reaching correlation coefficient values > 0.6 around 30 ns and peaking after 50 ns (Figure 3C). Similar results were observed for other CRBN residues near the dBET70 linker (Supplementary Figure SI7I,J). These results outline a continuous residue-to-residue communication pathway, such as the pathway progressing from Arg9 through Val866 to Ile2511 (Figure 3), that traces motion from the DDB1–CUL4A central region through DDB1 and into the CRBN–BRD4 interface (Figure 2A). Corresponding analyses from the second independently initialized trajectory reproduce similar lag-dependent trends (Supplementary Figure SI8). The observed propagation sequence mirrors the structural organization of the substrate-recognition module, which functions as a flexible mechanical unit connecting the CRL4A scaffold to the E3 ligase receptor, CRBN.

The culmination of this propagation cascade at ∼250 ns was further supported by distance analyses showing the formation of stabilizing contacts between CRBN residues and the dBET70 PROTAC linker. The hydrogen-bond distance between the side-chain of His2496 and its corresponding linker acceptor decreased from >3 Å at 0 ns to 1.8 Å after 250 ns, and the hydrogen-bond distance between the Asn2469 side-chain donor and its interacting linker acceptor decreased from >6 Å to <3 Å after 250 ns (Figure 2C). Additionally, a potential π – π stacking interaction was observed between Trp2498 and the linker’s aromatic ring, with the rings’ centroid distances decreasing to ∼3.5 Å at 250 ns (Figure 2C). These interactions reinforce the communication pathways observed in the TDTAC analyses and likely stabilize the degradation-competent conformation of the complex. Altogether, these observations demonstrate that motions at the DDB1–CUL4A central region are associated with a coherent propagation of motion through DDB1, culminating in structural tightening of CRBN residues near the dBET70 linker (Figure 2A). This mechanically coordinated cascade provides a quantitative framework for understanding how pivot flexibility may contribute to enhanced CRBN–dBET70–BRD4 engagement, thereby facilitating efficient targeted protein degradation.

### 3.3 Propagation of motion through the catalytic module: from CUL4A to RBX1

**FIGURE 4:**
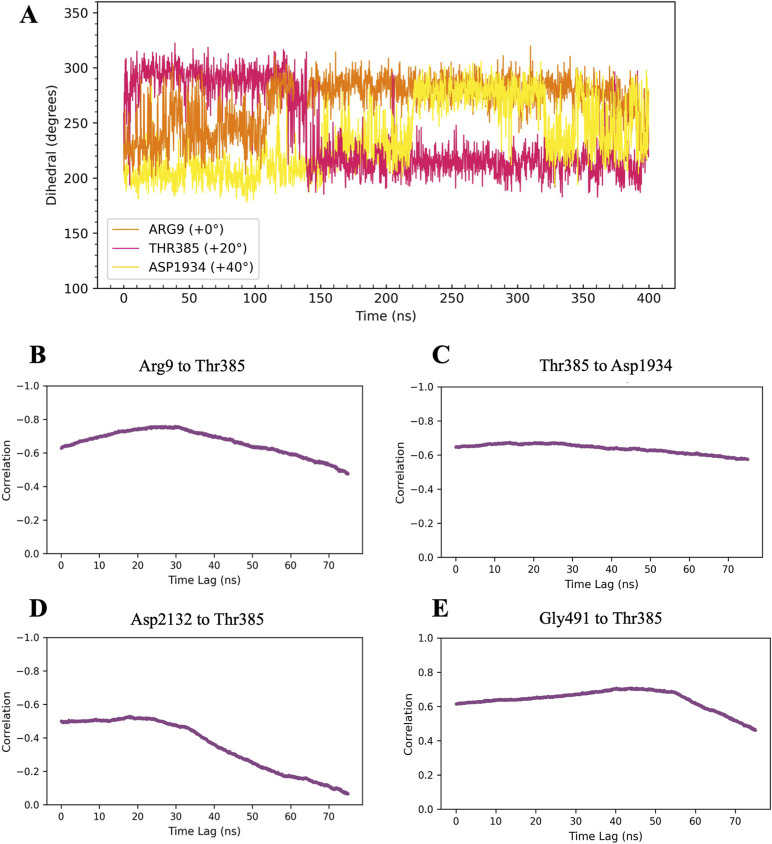
Time-dependent torsional angle correlations (TDTAC) within the catalytic module. **(A)** φ dihedral time series for Arg9, Thr385, and Asp1934. **(B)** TDTAC plot for propagation from Arg9 to Thr385. **(C)** TDTAC plot for propagation from Thr385 to Asp1934. **(D)** TDTAC plot for propagation from Asp2132 (E2) to Thr385 (CUL4A). **(E)** TDTAC plot for propagation from Gly491 to Thr385 within the CUL4A C-terminal region.

Analogous to the substrate-recognition module, the same DDB1–CUL4A central residues (Arg9, Val1090, Val1185, Asn1295, and Ser1316 (Figure 1B and SI4A)) that displayed early fluctuations (within the first 100 ns) also exhibited strong time-lagged correlations with residues in the distal CUL4A C-terminal interface–Ala384, Thr385, Gly444, and Phe447– which showed coordinated fluctuations around 150 ns (Supplementary Figure SI4D). A representative example is the TDTAC plot between the Arg9 φ dihedral (DDB1–CUL4A central region) and the Thr385 φ dihedral (CUL4A C-terminal interface), which showed a pronounced delayed correlation, with the correlation coefficient peaking at 0.7 after a lag of 30 ns (Figure 4B). When the Thr385 dihedral is shifted left by ∼30 ns, its fluctuations become closely aligned with Arg9 (Supplementary Figure SI6A). Similar time-lagged correlations were observed between other DDB1–CUL4A central residues and CUL4A C-terminal residues (Supplementary Figure SI9A–D). These results are consistent with early pivot-associated motions preceding subsequent conformational changes within the distal CUL4A C-terminal region.

The propagation of correlated motion extends further toward RBX1, near its interface with Ub. CUL4A C-terminal residues exhibit delayed correlations with RBX1 residues, including Thr1927, Gln1929, Val1930, Asp1934, and Arg1936 (Figure 1B). Coordinated fluctuations of these RBX1 residues in the later stages of the simulation (∼200–300 ns) are associated with repositioning of the Ub moiety relative to the E2 active site (Supplementary Figure SI4E). The TDTAC plots highlight this relationship; for example, the Thr385 φ dihedral maintains a correlation coefficient >0.6 with Asp1934 of RBX1 across the first 50 ns of lag time (Figure 4C). Comparable TDC relationships were observed for other residues near the RBX1–Ub interface (Supplementary Figure SI9E–I). These lag-dependent patterns are also seen in the second independently initialized trajectory, supporting consistent catalytic-module communication behavior (Supplementary Figure SI10).

Together, these findings reveal a continuous, time-ordered pattern of correlated motion along a residue-to-residue communication pathway–exemplified by the progression from Arg9 through Thr385 to Asp1934 (Figure 4A) that is consistent with signal transmission from the DDB1–CUL4A central region through CUL4A and into RBX1 (Figure 2A). This correlation sequence follows the structural organization of the catalytic module, which functions as a flexible scaffold linking central and distal catalytic regions. Distance analyses further support the functional relevance of these dynamics, showing that by ∼300 ns, the amino group of BRD4 Lys2603 and the C-terminal carbonyl oxygen of Ub Gly2016 approach distances of <2.5 Å, consistent with a catalytically competent geometry for isopeptide bond formation during ubiquitination (Wu et al., 2024) (Figure 2C). Overall, these observations suggest that motions originating near the DDB1–CUL4A central region are associated with coordinated, time-delayed rearrangements across the catalytic module, culminating in configurations compatible with catalysis. This propagation of correlated motion provides a quantitative framework for understanding how dynamic allosteric communication may facilitate efficient ubiquitin transfer in the dBET70-induced CRL4A E3 ligase assembly.

#### 3.3.1 Mechanically coupled motion and feedback propagation in the catalytic module

Dihedral analyses further revealed that residues at the E2 interface with BRD4, specifically Asn2130 and Asp2132, displayed early motions within the first 100 ns, coinciding with initial fluctuations observed at the central DDB1–CUL4A region (Supplementary Figure SI11A). Notably, Asp2132 is critical for catalysis, as previous structural studies have shown that its side-chain must approach BRD4 Lys2603 closely to facilitate charge transfer and enable isopeptide bond formation (Lv et al., 2021; Dixon et al., 2022). In the present simulation, the Asp2132–Gly2016 distance reached <6 Å at ∼300 ns, consistent with a catalytically competent configuration (Figure 2C). These E2 residues also exhibited delayed correlations with the CUL4A C-terminal region (Supplementary Figure SI12A,B). For example, the TDTAC profile revealed a strong correlation between Asp2132 and Thr385 within a 30 ns lag time, with an absolute correlation coefficient >0.5 (Figure 4D). When the Asp2132 dihedral motion around 110 ns was temporally aligned with the delayed Thr385 motion near 150 ns, their dihedral trajectories became highly coherent; outside this alignment window, the correlation decreased, consistent with loss of temporal synchrony (Supplementary Figure SI6B). This relationship suggests an additional propagation pathway (extending from Asp2132 through Thr385 and into Asp1934) that is consistent with communication from the E2–BRD4 interface through the CUL4A C-terminal region and into RBX1 (Figure 2B). These observations suggest that conformational fluctuations at the E2–BRD4 interface may contribute, alongside those at the DDB1-CUL4A central region, to coordinated downstream rearrangements within the catalytic module. Additionally, other CUL4A C-terminal residues–including Gly491, Tyr492, and Lys521– displayed early dihedral motions similar to those observed in the central DDB1–CUL4A region (Figure 4E and Supplementary Figure SI11B). These residues also exhibited time-lagged correlations with other CUL4A C-terminal residues (i.e., Ala384, Thr385, Gly444, and Phe447), which in turn are correlated with RBX1 residues that engage Ub (Supplementary Figure SI12C–E). Collectively, these observations indicate that multiple regions within the catalytic module participate in coordinated conformational dynamics, forming part of a time-lagged correlation network associated with positioning RBX1 and Ub into catalytically relevant configurations around 300 ns.

The synchronized structural rearrangements of the DDB1–CUL4A central region and residues within the catalytic module are consistent with coordinated, rather than independent, motions. This behavior suggests the presence of feedback-like interactions across the CRL4A E3 ligase assembly, wherein early fluctuations within E2 and the CUL4A C-terminal region are associated with subsequent propagation of conformational changes toward RBX1. The TDTAC analysis quantitatively supports this dynamic coordination, where early dihedral shifts occur within the first 100 ns, but optimal catalytic alignment, assessed by the Lys2603–Gly2016 and Asp2132–Lys2603 distances, is observed later in the trajectory (at ∼300 ns), following reorientation of RBX1 residues (Figure 2D). Together, these findings reveal a time-ordered network of correlated motions within the catalytic module, in which local catalytic-site fluctuations and global pivot-associated dynamics are interconnected. This temporally organized coordination provides a quantitative framework for understanding how dynamic communication across the assembly may facilitate efficient ubiquitin transfer in the dBET70-induced CRL4A E3 ligase assembly.

### 3.4 Coordinated bidirectional motions between the substrate-recognition and catalytic modules and functional applications

While motion propagation can be traced independently through the substrate-recognition and catalytic modules, dihedral analyses indicate that both modules exhibit temporally coordinated fluctuations, suggesting that dynamics in the central DDB1–CUL4A region are associated with a coherent dual-pathway response (Figure 2A). The observed lag times across both propagation pathways provide a quantitative measure of the timescale of information transfer within this multi-protein assembly. These results suggest that the substrate-recognition and catalytic modules do not behave as independent structural domains, but instead exhibit coordinated dynamical responses across shared temporal regimes. The TDTAC analyses indicate that the central DDB1–CUL4A region acts as an early dynamic hub, initiating correlated motions during the early stages of the simulation (∼100 ns). This is followed by coordinated fluctuations in both the DDB1–CRBN interface-proximal region and the CUL4A C-terminal region at intermediate stages of the simulation (∼150 ns). Subsequent propagation towards the CRBN–BRD4 interface and RBX1–Ub interface indicates that the central region serves as a mechanical relay, associated with the redistribution of correlated motions across the assembly and contributing to conformational changes consistent with ubiquitination-competent geometries (Figure 2). This temporally ordered behavior suggests that flexibility at the DDB1-CUL4A central region is associated with long-range coordination of domain motions across the CRL4A E3 ligase assembly. Both independent trajectories show consistent temporal ordering and conserved propagation regions, with some residue-level variations but preserving the overall communication architecture.

Collectively, these results highlight the utility of the TDTAC framework for resolving time-lagged propagation of dihedral correlations in the dBET70-induced CRL4A E3 ligase complex. In contrast to conventional RMSD or static correlation analyses, which provide time-averaged or non-directional metrics, TDTAC captures directional, lag-resolved relationships between residue motions. The observation of concurrent propagation along multiple pathways (Figures 2A,B) suggests a coordinated network of residue-level communication that may contribute to the stabilization of ubiquitination-competent conformations. These findings support the interpretation that pivot regions function as dynamic hubs that coordinate global conformational rearrangements across the assembly. In this framework, the DDB1–CUL4A central region acts as a primary dynamic relay, translating localized fluctuations into system-wide correlated responses, thereby facilitating coordinated motion across the CRL4A ubiquitination machinery and enabling efficient PROTAC-mediated protein degradation.

## 4 Conclusion

Proteins depend on coordinated, time-dependent motions to perform biological functions, yet conventional trajectory analyses often lack the ability to resolve how these dynamics propagate across multi-protein assemblies. This study introduces a generalized TDTAC framework that incorporates residue-level lag times to quantitatively map directional, sequential correlations across molecular trajectories. By using torsional angles as primary degrees of freedom, TDTAC preserves intrinsic rotational dynamics and provides a more physically grounded representation of residue fluctuations compared with Cartesian-based analyses (Chang and Gilson, 2003; Ghosh and Vishveshwara, 2007; Das et al., 2016).

Application of the TDTAC framework to dBET70-induced structural dynamics within the CRL4A E3 ligase complex reveals a structured propagation network of time-lagged correlations. Motions originating at the central DDB1–CUL4A region propagate along two dominant pathways spanning the substrate-recognition and catalytic modules. These pathways are associated with coordinated time-lagged rearrangements that adopt conformations consistent with ubiquitination-competent geometries during later stages of the simulation. Nearly synchronous fluctuations across both pathways suggest that the central region plays a key role in distributing conformational changes throughout the assembly. These propagation patterns and temporal relationships are consistently observed across independently initialized trajectories, supporting the robustness of the identified communication network.

Collectively, these findings establish a quantitative framework linking local structural flexibility to distal functional sites through time-lagged motion propagation. The TDTAC framework provides a generalizable and computationally efficient approach for investigating dynamic communication, allosteric signaling, and interdomain coordination in biomolecular complexes. By resolving temporal propagation of conformational fluctuations, TDTAC enables detailed characterization of dynamical communication networks in biomolecular assemblies and provides a basis for interpreting coordinated protein motion in complex systems.

## Data Availability

The original contributions presented in the study are included in the article/Supplementary Material, further inquiries can be directed to the corresponding author.

## References

[B1] Ai R. Fatmi Q. Chang C. A. (2010). T-Analyst: a program for efficient analysis of protein conformational changes by torsion angles. J. Comput. Aided Mol. Des. 24 (10), 819–827. 10.1007/s10822-010-9376-y 20689979 PMC2940022

[B2] Albert R. Jeong H. Barabási A.-L. (1999). Diameter of the world-wide web. Nature 401 (6749), 130–131. 10.1038/43601

[B3] Bastidas O. H. Sevarac Z. (2024). Time dependent dihedral angle oscillations of the spike protein of SARS-COV-2 reveal favored frequencies of dihedral angle rotations. Nat. Sci. Rep. 14 (3323), 3323. 10.1038/s41598-024-53954-9 38336854 PMC10858279

[B4] Békés M. Langley D. R. Crews C. M. (2022). PROTAC targeted protein degraders: the past is prologue. Nat. Rev. Drug Discov. 21 (3), 181–200. 10.1038/s41573-021-00371-6 35042991 PMC8765495

[B5] Branden C. Tooze J. (1991). Introduction to Protein Structure. First Edition. New York, NY: Garland Publishing.

[B6] Bricelj A. Steinebach C. Kuchta R. Gütschow M. Sosič I. (2021). E3 ligase ligands in successful PROTACs: an overview of syntheses and linker attachment points. Front. Chem. 9, 707317. 10.3389/fchem.2021.707317 34291038 PMC8287636

[B7] Briki F. Genest D. (1994). Canonical analysis of correlated atomic motions in DNA from molecular dynamics simulation. Biophys. Chem. 52 (1), 34–43. 10.1016/0301-4622(94)00063-8 7948709

[B8] Chang C. A. Gilson M. K. (2003). Tork: conformational analysis method for molecules and complexes. J. Comput. Chem. 24 (16), 1987–1998. 10.1002/jcc.10325 14531053

[B9] Chang C. A. Potter M. Gilson M. K. (2003). Calculation of molecular configuration integrals. J. Phys. Chem. B 107 (4), 1048–1055. 10.1021/jp027149c

[B10] Chong S. Ham S. (2021). Time-dependent communication between multiple amino acids during protein folding. Chem. Sci. 12 (16), 5944–5951. 10.1039/d0sc07025d 35342544 PMC8871807

[B11] Dale B. Cheng M. Park K.-S. Kaniskan H. Ü. Xiong Y. Jin J. (2021). Advancing targeted protein degradation for cancer therapy. Nat. Rev. Cancer 21 (10), 638–654. 10.1038/s41568-021-00365-x 34131295 PMC8463487

[B12] Das A. Ghosha M. Chakrabarti J. (2016). Time dependent correlation between dihedral angles as probe for long range communication in proteins. Chem. Phys. Lett. 645, 200–204. 10.1016/j.cplett.2015.12.060

[B13] Dixon T. MacPherson D. Mostofian B. Dauzhenka T. Lotz S. McGee D. (2022). Predicting the structural basis of targeted protein degradation by integrating molecular dynamics simulations with structural mass spectrometry. Nat. Commun. 13 (1), 5884. 10.1038/s41467-022-33575-4 36202813 PMC9537307

[B14] Dror R. O. Dirks R. M. Grossman J. P. Xu H. Shaw D. E. (2012). Biomolecular simulation: a computational microscope for molecular biology. Annu. Rev. Biophysics 41, 429–452. 10.1146/annurev-biophys-042910-155245 22577825

[B15] Dutta S. Ghosh M. Chakrabarti J. (2017). Spatio-temporal coordination among functional residues in protein. Sci. Rep. 7, 40439. 10.1038/srep40439 28091537 PMC5238388

[B16] Filippakopoulos P. Qi J. Picaud S. Shen Y. Smith W. B. Fedorov O. (2010). Selective inhibition of BET bromodomains. Nature 468 (7327), 1067–1073. 10.1038/nature09504 20871596 PMC3010259

[B17] Gamier N. Genest M. (1996). Correlated motions and propagation of the effect of a local conformational change in the transmembrane helix of the c-e&B2 encoded protein and in its V659E mutant, studied by molecular dynamics simulations. Biophys. Chem. 58 (3), 225–237. 10.1016/0301-4622(95)00106-9 8820408

[B18] Genest D. (1996). How long does DNA keep the memory of its conformation? A time dependent canonical correlation analysis of molecular dynamics simulation. Biopolymers 38 (3), 389–399. 10.1002/(SICI)1097-0282(199603)38:3<389::AID-BIP11>3.0.CO;2-8 8906974

[B19] Ghosh A. Vishveshwara S. (2007). A study of communication pathways in methionyl-tRNA synthetase by molecular dynamics simulations and structure network analysis. Proc. Natl. Acad. Sci. U.S.A. 104 (40), 15711–15716. 10.1073/pnas.0704459104 17898174 PMC2000407

[B20] Goodey N. Benkovic S. (2008). Allosteric regulation and catalysis emerge *via* a common route. Nat. Chem. Biol. 4 (8), 474–482. 10.1038/nchembio.98 18641628

[B21] He X. Man V. H. Yang W. Lee T. S. Wang J. (2020). A fast and high-- quality charge model for the next generation general AMBER force field. J. Chem. Phys. 153, 114502. 10.1063/5.0019056 32962378 PMC7728379

[B22] Jenkins G. M. Watts D. G. (1968). Spectral Analysis and its Applications. San Francisco, CA: Holden Day.

[B23] Johnson Q. R. Lindsay R. J. Shen T. (2018). CAMERRA: an analysis tool for the computation of conformational dynamics by evaluating residue-residue associations. J. Comput. Chem. 39 (20), 1568–1578. 10.1002/jcc.25192 29464733

[B24] Kessel A. Ben-Tal N. (2018). Introduction to Proteins: Structure, Function, and Motion. Second Edition. Boca Raton, FL: Chapman and Hall/CRC Press.

[B25] Lange O. F. Grubmüller H. (2006). Generalized correlation for biomolecular dynamics. Proteins 62 (4), 1053–1061. 10.1002/prot.20784 16355416

[B26] Lee J. Lee Y. Jung Y. M. Park J. H. Yoo H. S. Park J. (2022). Discovery of E3 ligase ligands for target protein degradation. Molecules 27 (19), 6515. 10.3390/molecules27196515 36235052 PMC9573645

[B27] Liu Z. Hu M. Yang Y. Du C. Zhou H. Liu C. (2022). An overview of PROTACs: a promising drug discovery paradigm. Mol. Biomed. 3, 46. 10.1186/s43556-022-00112-0 36536188 PMC9763089

[B28] Loutchko D. Flechsig H. (2020). Allosteric communication in molecular machines *via* information exchange: what can be learned from dynamical modeling. Biophys. Rev. 12, 443–452. 10.1007/s12551-020-00667-8 32198636 PMC7242553

[B29] Lu J. Qian Y. Altieri M. Dong H. Wang J. Raina K. (2015). Hijacking the E3 ubiquitin ligase cereblon to efficiently target BRD4. Chem. Biol. 22 (6), 755–763. 10.1016/j.chembiol.2015.05.009 26051217 PMC4475452

[B30] Lv D. Pal P. Liu X. Jia Y. Thummuri D. Zhang P. (2021). Development of a BCL-XL and BCL-2 dual degrader with improved anti-leukemic activity. Nat. Commun. 12 (1), 1–14. 10.1038/s41467-021-27210-x 34824248 PMC8617031

[B31] Maier J. A. Martinez C. Kasavajhala K. Wickstrom L. Hauser K. E. Simmerling C. (2015). ff14SB: improving the accuracy of protein side chain and backbone parameters from ff99SB. J. Chem. Theory Comput. 11, 3696–3713. 10.1021/acs.jctc.5b00255 26574453 PMC4821407

[B32] Melvin R. L. Xiao J. Berenhaut K. S. Godwin R. C. Salsbury F. R. (2018). Using correlated motions to determine sufficient sampling times for molecular dynamics. Phys. Rev. E. 98 (2), 023307. 10.1103/PhysRevE.98.023307 30253618 PMC6325644

[B33] Nussinov R. Liu Y. Zhang W. Jang H. (2023). Protein conformational ensembles in function: roles and mechanisms. RSC Chem. Biol. 4 (11), 850–864. 10.1039/d3cb00114h 37920394 PMC10619138

[B34] Qi S. M. Dong J. Xu Z. Y. Cheng X. D. Zhang W. D. Qin J. J. (2021). PROTAC: an effective targeted protein degradation strategy for cancer therapy. Front. Pharmacol. 12, 692574. 10.3389/fphar.2021.692574 34025443 PMC8138175

[B35] Qin C. Hu Y. Zhou B. Fernandez-Salas E. Yang C.-Y. Liu L. (2018). Discovery of QCA570 as an exceptionally potent and efficacious proteolysis targeting chimera (PROTAC) degrader of the bromodomain and extra-terminal (BET) proteins capable of inducing complete and durable tumor regression. J. Med. Chem. 61 (15), 6685–6704. 10.1021/acs.jmedchem.8b00506 30019901 PMC6545111

[B36] Ravasz E. Somera A. L. Mongru D. A. Oltvai Z. N. Barabási A. L. (2002). Hierarchical organization of modularity in metabolic networks. Science 297 (5586), 1551–1555. 10.1126/science.1073374 12202830

[B37] Schreiber T. (2000). Measuring information transfer. Phys. Rev. Lett. 85 (2), 461–464. 10.1103/PhysRevLett.85.461 10991308

[B38] Sethi A. Eargle J. Black A. A. Luthey-Schulten Z. (2009). Dynamical networks in tRNA:protein complexes. Proc. Natl. Acad. Sci. U.S.A. 106 (16), 6620–6625. 10.1073/pnas.0810961106 19351898 PMC2672494

[B39] Tang Z. Chang C. A. (2017). Systematic dissociation pathway searches guided by principal component modes. J. Chem. Theory Comput. 13 (5), 2230–2244. 10.1021/acs.jctc.6b01204 28418661 PMC5920795

[B40] Winter G. E. Buckley D. L. Paulk J. Roberts J. M. Souza A. Dhe-Paganon S. (2015). Phthalimide conjugation as a strategy for *in vivo* target protein degradation. Science 348 (6241), 1376–1381. 10.1126/science.aab1433 25999370 PMC4937790

[B41] Wodak S. J. Paci E. Dokholyan N. V. Berezovsky I. N. Horovitz A. Li J. (2019). Allostery in its many disguises: from theory to applications. Cell. Struct. 27 (4), 566–578. 10.1016/j.str.2019.01.003 30744993 PMC6688844

[B42] Wu K. Y. Hung T. I. Chang C. A. (2024). PROTAC-induced protein structural dynamics in targeted protein degradation. eLife 13, RP101127. 10.7554/eLife.101127 40014381 PMC11867615

[B43] Yu H. Dalby P. A. (2020). A beginner’s guide to molecular dynamics simulations and the identification of cross-correlation networks for enzyme engineering. Methods Enzymol. 643, 15–49. 10.1016/bs.mie.2020.04.020 32896280

[B44] Zhou B. Hu J. Xu F. Chen Z. Bai L. Fernandez-Salas E. (2018). Discovery of a small-molecule degrader of bromodomain and extra-terminal (BET) proteins with picomolar cellular potencies and capable of achieving tumor regression. J. Med. Chem. 61 (2), 462–481. 10.1021/acs.jmedchem.6b01816 28339196 PMC5788414

