# Supplementary material for: TDTAC: a generalized time-dependent torsion angle correlation framework for resolving directional coordination in large biomolecular assemblies
Source: Front Mol Biosci. 2026 Jun 3;13:1821703. doi: 10.3389/fmolb.2026.1821703 (PMC13271675; doi:10.3389/fmolb.2026.1821703)
Supplement: Supplementary file 1 [file DataSheet1.pdf]

Supplemental Information

TDTAC: A Generalized Time-Dependent Torsion Angle Correlation Framework for Resolving Directional Coordination in Large Biomolecular Assemblies

Carolina Escobar Palacio[1], Tongye Shen[2], Chia-En A. Chang[1*].

[1]Department of Chemistry, University of California, Riverside.
[2]Department of Biochemistry and Cellular and Molecular Biology, University of Tennessee, Knoxville

*** Correspondence:**
Chia-En A. Chang
chiaenc@ucr.edu

1. Methods

1.1 Theory and Equations of Time-Dependent Torsional Angle Correlation (TDTAC)

Traditional Pearson correlation analysis quantifies the linear relationship between two variables, X and Y, across N time points but assumes simultaneous motion[1]. To account for the temporal propagation of correlated motions, the Pearson formulation was modified to include an explicit time-lag parameter, L, enabling evaluation of how fluctuations in one variable influence another at a later time. The resulting TDC equation is expressed as:

$$TDC_{X,Y}(L) = \frac{\sum_{i=1}^{N-m} X_i' \, Y_{i+L}'}{\sqrt{\sum_{i=1}^{N-m} (X_i')^2 \, * \, \sum_{i=1}^{(N-m)+L} (Y_{i+L}')^2}} \qquad \textbf{(Eq 1)}$$

Where $X_i' = (X_i - \bar{X})$ and $Y_{i+L}' = (Y_{i+L} - \bar{Y})$ are the mean-centered values of the selected degrees of freedom (i.e. X or Y) at time i, N is the total number of frames in the MD trajectory, and L is the applied time lag ranging from zero to a defined maximum m. The maximum lag m is chosen to ensure sufficient overlap between the truncated time series, thereby maintaining statistically meaningful sampling and preventing unreliable correlations at large lag times. The calculation of TDC is inherently direction dependent. For lags greater than zero, the correlation is asymmetric, i.e., $TDTAC_{X,Y}(L > 0) \neq TDTAC_{Y,X}(L > 0)$. This asymmetry arises because the first variable X is held fixed, while the second variable Y is incrementally shifted by the lag up to the maximum value, capturing the directional propagation of correlated motion.

The formulation of Eq. 1 preserves the normalization properties of the Pearson coefficient ($-1 \leq$ TDTAC ≤ 1), allowing direct comparison of correlation magnitudes across time lags. Importantly, the time-shifted approach enables generation of various TDTAC maps that represent residue–residue correlations as a continuous function of lag time and allows visualization of both instantaneous and delayed communication events within a protein.

1.2 Circular Statistics for Dihedral Data

Residue-level internal motions were quantified using backbone ϕ dihedral angles extracted from MD trajectories. Because dihedral angles are circular variables, linear arithmetic can introduce artifacts near the periodic boundaries (0°/360° and ±180°). To preserve angular periodicity, trigonometric representations based on $\sin\theta$ and $\cos\theta$ were used for circular averaging and subtraction (Eq. 2 and 3) when constructing the correlation matrices from dihedral data. Mean and subtraction referred to in Eq. 1 are the corresponding circular operations when we deal with torsions. The resulting values were reconstructed into angular form using the two-argument arctangent function (arctan2), preserving the correct phase representation of dihedral angles and avoiding boundary discontinuities[2]. Correlations between residue pairs were calculated on the transformed circular data using Eq. 1, effectively capturing phase-synchronous fluctuations rather than linear deviations and correctly representing the periodic character of torsional motions.

$$tan\left(\frac{X_1 + X_2 + \ldots + X_N}{N}\right) = \frac{sin(X_1) + sin(X_2) + \ldots + sin(X_N)}{cos(X_1) + cos(X_2) + \ldots + cos(X_N)} \quad \textbf{(Eq 2)}$$

$$tan(X - Y) = \frac{sin(X)\,cos(Y) - sin(Y)\,cos(X)}{cos(X)\,cos(Y) + sin(X)\,sin(Y)} \quad \textbf{(Eq 3)}$$

1.3 Implementation and Computational Framework

The TDTAC equation was implemented in Python 3.9. The algorithm reads 2D arrays where each column represents a residue's dihedral time series and iteratively computes the time-dependent correlation between all residue pairs across a defined set of time lags. The resulting output is a rank-3 tensor M of dimension $(L \times i \times i)$, with each slice corresponding to a correlation matrix at a given lag L and residue number i. Because the resulting tensor M contains on the order of 10^9 elements for $L = 50$ns and $i = 2664$ residues, computations were vectorized to ensure computational efficiency.

Numerical validation of the TDTAC equation in Eq. 1 was verified using small, controlled datasets. This was done by comparing TDTAC results with those obtained from the classical autocorrelation function (for same-variable comparisons) and from the Pearson correlation (for zero-lag cases)[3,4,5]. At zero lag, TDTAC is equivalent to the Pearson correlation computed on the same dihedral time series, providing a direct baseline for comparison with standard equilibrium-based correlation analysis. Agreement among these methods confirmed that the TDTAC algorithm reliably handles both linear and circular variables, reproducing known statistical relationships, and establishing a robust foundation for subsequent application to protein systems.

1.4 Molecular Dynamics and Dihedral Analysis of the dBET70 Degradation Machinery Complex

1.4.1. MD Simulation

The dBET70-induced degradation complex (comprising CRBN, DDB1, CUL4A, NEDD8, RBX1, E2, Ub, and BRD4) was constructed and simulated using the AMBER20 software package, as described in a previous study by Wu, et al. (2024)[6]. The system was parameterized with the ff14SB force field[7] for proteins and GAFF2[8] for the dBET70 ligand. The system was

solvated in an explicit water box with distance 12 Å from the edge of the protein complex. TIP3P[9] water model was used and the system was neutralized with counterions.

Sequential energy minimizations were performed for hydrogen atoms, side chains, and the full complex to remove steric clashes. This was followed by 3 ns of NPT equilibration. Long-range electrostatics were treated using the Particle Mesh Ewald (PME) method[10], and all bonds involving hydrogens were constrained using SHAKE[11]. Production simulations were done for 400 ns under periodic boundary conditions at 300 K and 1 atm, using a 2-fs integration time step. Trajectory frames were saved every 50ps for analysis. VMD[12] was used for structural inspection, atomic distance calculations, and RMSD computations.

1.4.2. Dihedral Extraction and Hinge Motion Identification

Backbone φ dihedral data was extracted directly from the MD trajectory using the program T-Analyst[13], which computes torsional angles from Cartesian coordinates by evaluating the torsion defined by four sequentially bonded atoms according to the standard internal coordinate convention. The resulting time series, $\theta_i(t)$, generated for each selected residue is used as input for subsequent TDTAC calculations.

To identify potential hinge-like flexion points within the complex, relative RMSD analysis was used on the full MD trajectory. Prior to RMSD calculations of backbone Cα atoms, a specific protein region was selected to serve as the reference structure for aligning the trajectory. After visualizing the trajectories with VMD[12], rigid regions and potential pivot regions of the CRL4A E3 ligase scaffold were selected for trajectory alignment, including the central region between DDB1-CUL4A, the DDB1-CRBN interface, and the CUL4A C-terminal interface (Fig. 2). RMSD for BRD4 (the protein of interest) and the E2 enzyme (the Ub carrier and catalytic partner of the E3 ligase) was calculated relative to these anchor regions. This relative RMSD approach quantifies BRD4 and E2 motion with respect to the anchored distal regions within the full dBET70-bound CRL4A E3 ligase assembly, enabling detection of inter-domain flexibility and pivot regions that act as flexion points. The resulting RMSD profiles were analyzed for distinct transitions, which are interpreted as signatures of flexion events that mediate conformational coordination between BRD4, the dBET70 linker, and the CRL4A E3 ligase complex. Importantly, all TDTAC calculations were performed on backbone dihedral angles, which are internal coordinates and therefore invariant to global translational and rotational motion. As a result, trajectory superposition onto a reference frame does not affect the computed dihedral time series or the resulting correlation values. Structural alignment was only applied for RMSD-based analyses used to identify pivot regions and assess large-scale conformational transitions.

1.4.3. Network characterization of structure and dynamics

Beyond delineating specific important dynamic correlations between residues, it is important to examine the overall organization of coordinated motions within the complex. Network science has emerged as the leading analysis tool for analyzing complex dynamical systems by representing them as abstract graphs that capture essential dynamics at the global scale[14,15]. Here, a protein time-dependent correlation network (TDCN) is constructed so that each node represents an amino acid residue and each edge denotes a strong dynamic correlation link between pairs of residues. Specifically, once the rank-3 tensor M is constructed, a rank-2 matrix D of dimension $(i \times i)$ is obtained by selecting a specific non-zero lag index L, where i is the residue number.

Each selected matrix D_{ii} therefore corresponds to the residue-residue correlation matrix at a given lag-time L. This procedure preserves lag-specific correlation structure and enables analysis of time-delayed dynamic relationships between residue pairs.

Given the matrix D_{ii}, and a threshold parameter d, an adjacency matrix A_{ii} was constructed such that $A_{ii} = 1$ if $D_{ii} > d,$ or $A_{ii} = 0$ otherwise. The cutoff parameter d was selected to control the overall graph density, typically fixed to a defined fraction (e.g., 1%) of the complete network, enabling consistent comparison across systems. Once the adjacency matrix is constructed, network analyses were performed to evaluate and quantify node-based properties of such TDCN including clustering coefficients and centrality measurements[16–18]. These metrics quantify the extent of local coordination and the relative importance of residues in mediating long-range dynamic communication. To further characterize coordinated large-scale organization, community structure analysis was performed to identify modules that maximize network modularity, revealing groups of residues exhibiting strongly correlated torsional dynamics[19]. Additionally, a static protein structure network was also constructed, with nodes representing residues and edges defined based on spatial proximity in the average structure of the MD ensemble. This structural graph provides a baseline representation of residue contact topology against which the time-dependent dynamic network can be contrasted.

2. Conservation of TDTAC-Derived Communication Pathways Across Independent MD Trajectories.

To assess robustness, TDTAC and network analyses were repeated on an independent trajectory initialized with different random velocities. While residue-level participation differs between simulations, both trajectories consistently recover the same five functional regions: the DDB1–CUL4A central pivot, DDB1–CRBN interface-proximal region, CRBN–BRD4 interface, CUL4A C-terminal region, and RBX1–Ub interface (Fig. SI3). In both cases, early and intermediate propagation events map to the same structural interfaces, indicating conservation of the underlying communication architecture at the regional level (Fig. SI8 and SI10). The second trajectory additionally shows increased participation of BRD4 interface residues during late-stage propagation, suggesting enhanced engagement of the substrate-recognition module at the culmination of the cascade. Despite these differences, both trajectories exhibit highly similar lag-dependent correlation profiles, temporal ordering, and propagation timescales, confirming that the TDTAC-derived communication network is robust to stochastic initial conditions.

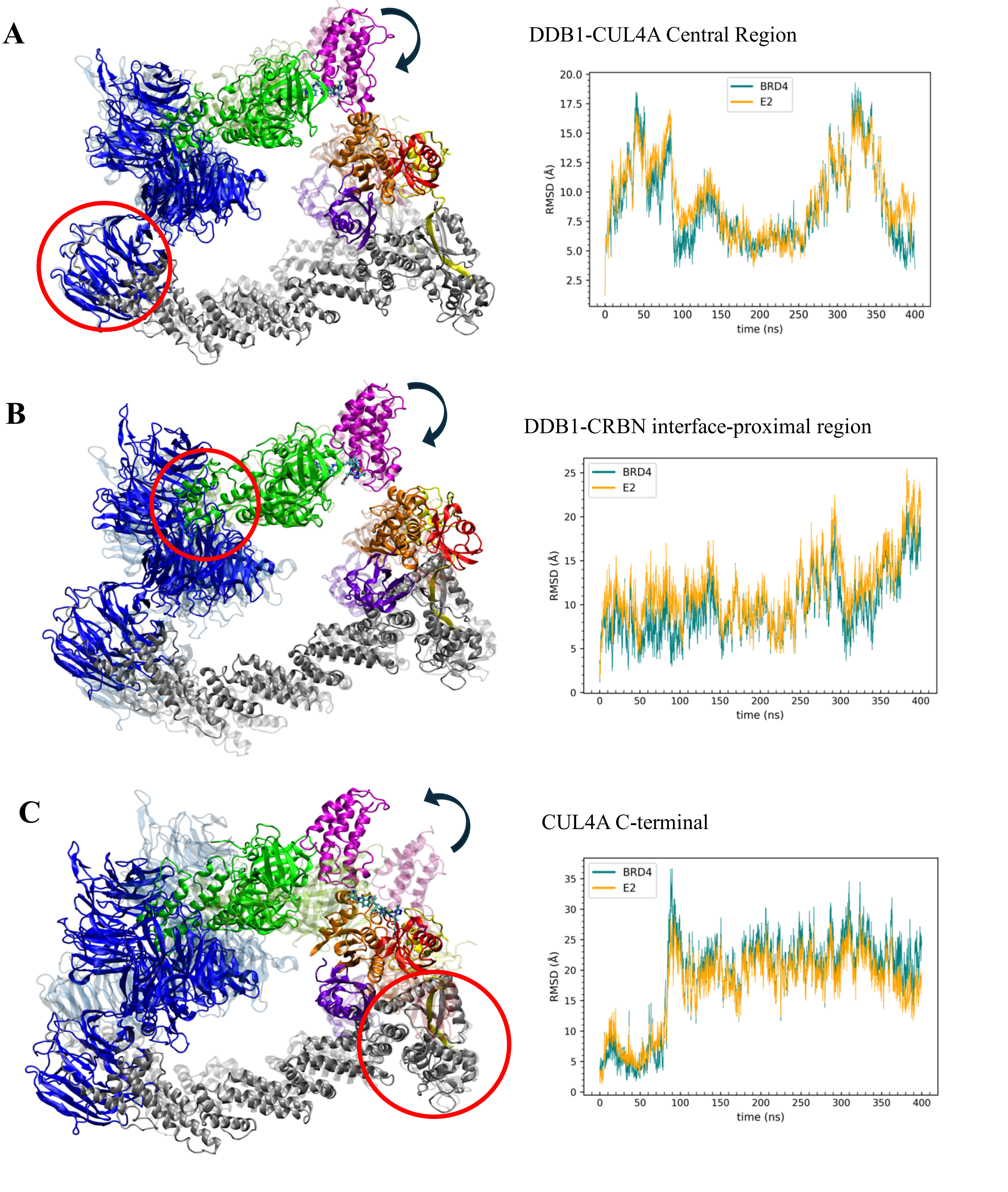

Supplemental Figure 1. Alignment structures and relative RMSD plots for BRD4 (teal) and E2 (orange). **(A)** RMSD relative to DDB1–CUL4A central region (red circle). Structures at 0 ns are shown in transparent representation; structures at ~320 ns are shown opaque. **(B)** RMSD relative to DDB1–CRBN interface-proximal region (red circle). Transparent and opaque representations correspond to 0 ns and ~360 ns, respectively. **(C)** RMSD relative to CUL4A C-terminal region (red circle). Transparent and opaque representations correspond to 0 ns and ~95 ns, respectively**.** For all panels, depicted arrows indicate direction of conformational changes from the initial (transparent) to later (opaque) structures.

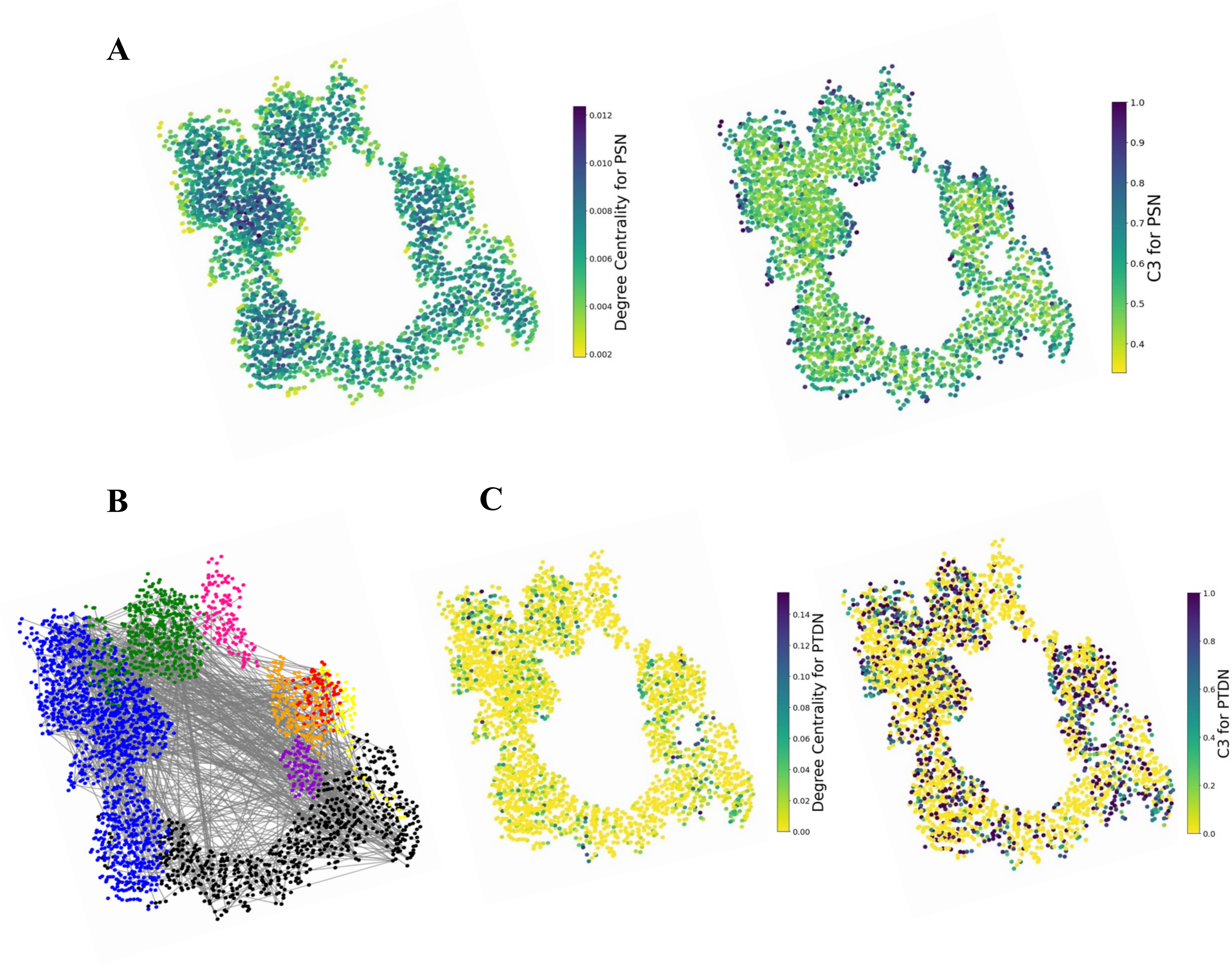

Supplemental Figure 2. Structural and dynamic torsional correlation networks computed from an independent MD trajectory (seed 1). **(A)** Static protein structure network (PSN) showing degree centrality and clustering coefficient C3. **(B)** Protein dynamic correlation network at a lag time of 50 ns, with CUL4A (gray), DDB1 (blue), NEDD8 (purple), RBX1 (yellow), E2 (orange), ubiquitin (red), CRBN (green), and BRD4 (magenta). **(C)** Protein torsional dynamic network (PTDN) at a 50 ns lag, showing degree centrality and clustering coefficient C3.

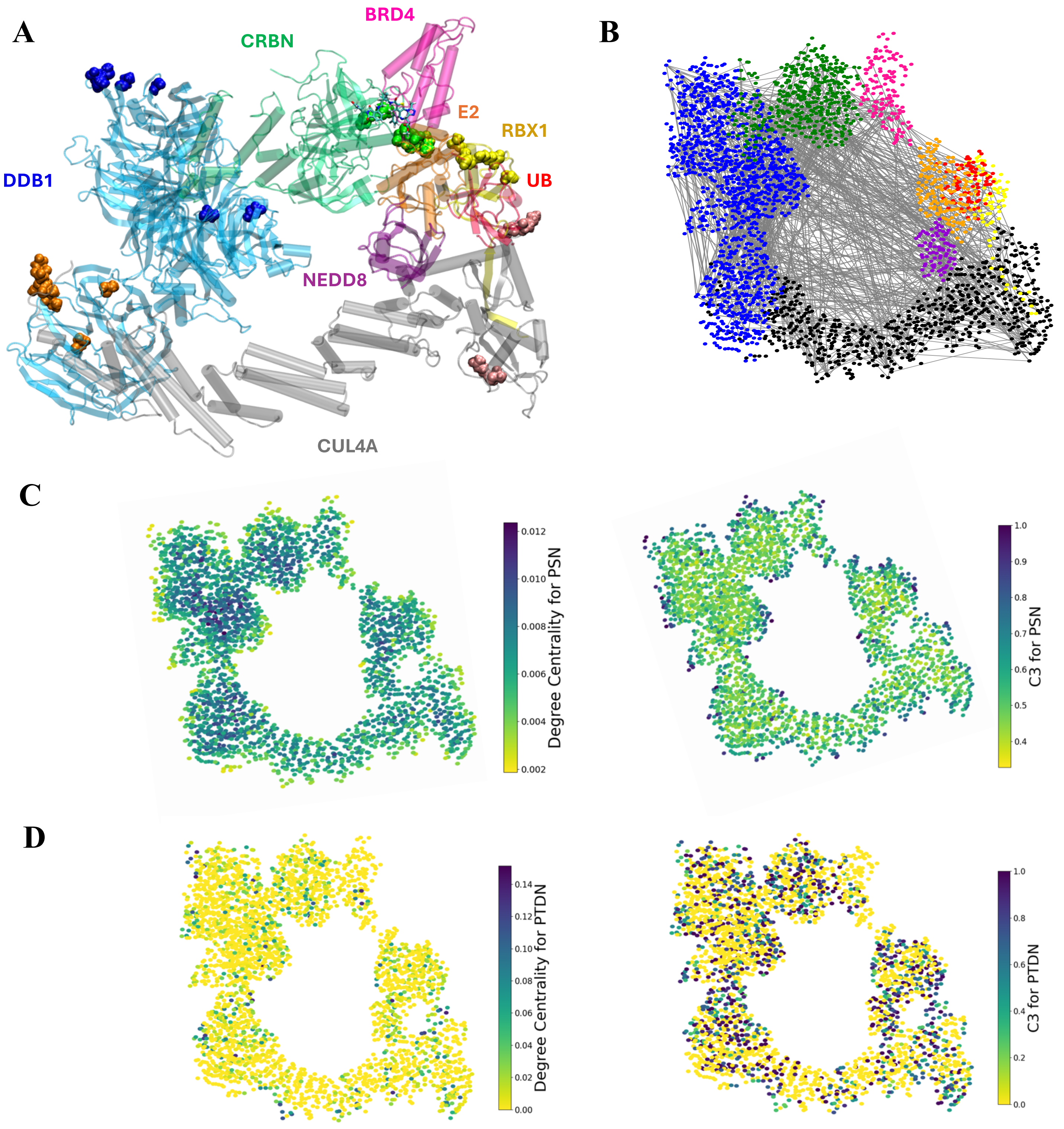

Supplemental Figure 3. Structural and dynamic torsional correlation networks computed from an independent MD trajectory initialized with a different random seed (seed 2). The PROTAC-mediated degradation complex is shown with subunits colored as follows: CUL4A (gray), DDB1 (blue), NEDD8 (purple), RBX1 (yellow), E2 (orange), ubiquitin (red), CRBN (green), and BRD4 (magenta). **(A)** Structural representation highlighting clusters of residues found at each pivot region; clustered residues are color-coded by interface: DDB1–CUL4A (orange), DDB1–CRBN (blue), CRBN–BRD4 (green), CUL4A C-terminus (pink), and RBX1–UB (yellow). **(B)** Protein dynamic correlation network at lag time of 50 ns. **(C)** Static protein structure networks (PSN) showing degree centrality and clustering coefficient (C3). **(D)** Protein torsional dynamic networks (PTDN) at a 50 ns lag, showing degree centrality and clustering coefficient C3.

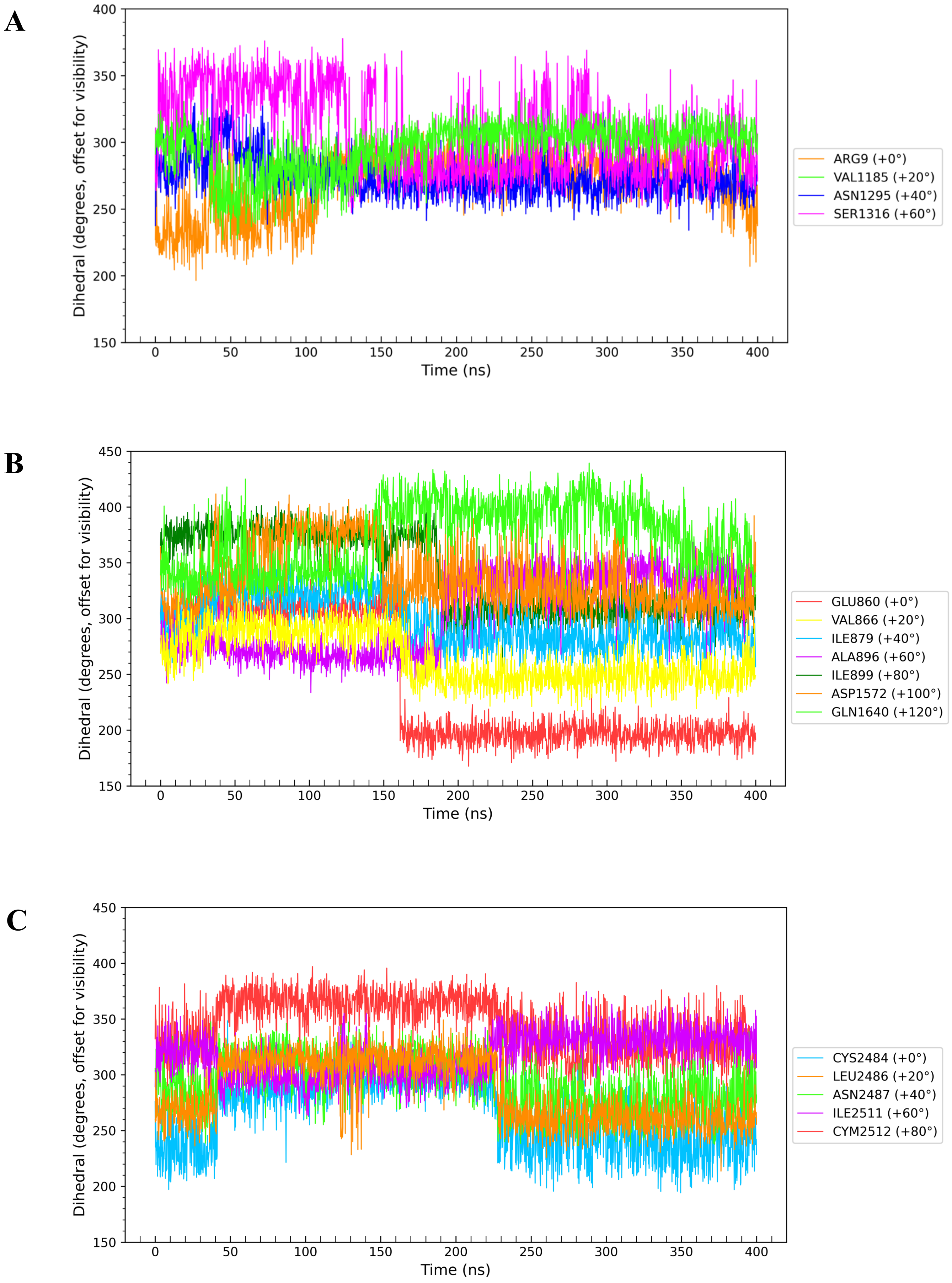
A
Dihedral (degrees, offset for visibility)
Time (ns)
ARG9 (+0°)
VAL1185 (+20°)
ASN1295 (+40°)
SER1316 (+60°)
B
Dihedral (degrees, offset for visibility)
Time (ns)
GLU860 (+0°)
VAL866 (+20°)
ILE879 (+40°)
ALA896 (+60°)
ILE899 (+80°)
ASP1572 (+100°)
GLN1640 (+120°)
C
Dihedral (degrees, offset for visibility)
Time (ns)
CYS2484 (+0°)
LEU2486 (+20°)
ASN2487 (+40°)
ILE2511 (+60°)
CYM2512 (+80°)

D

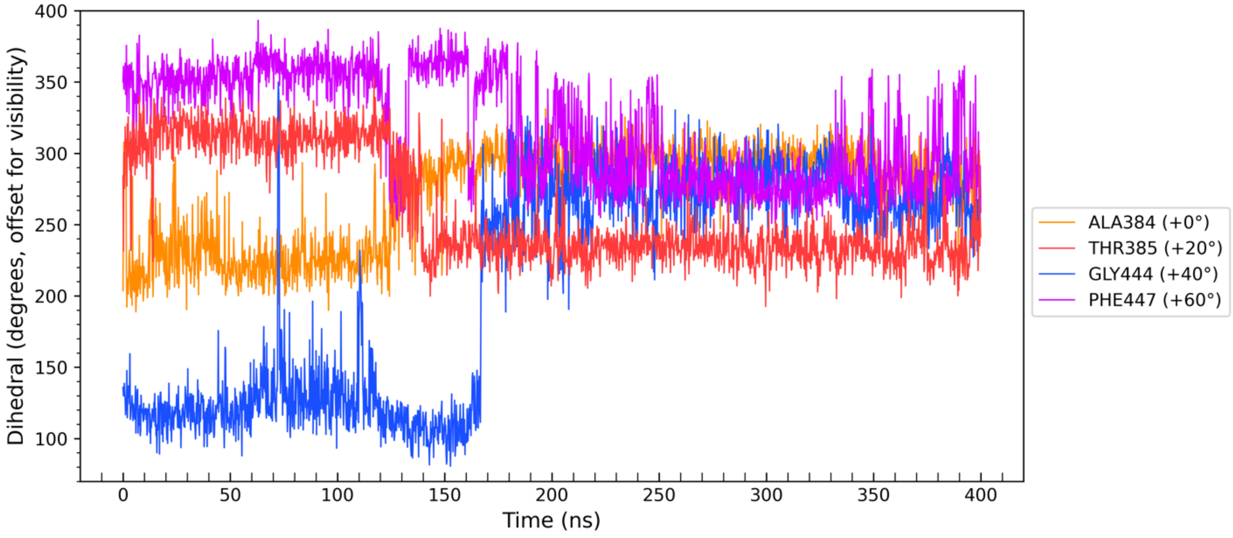

E

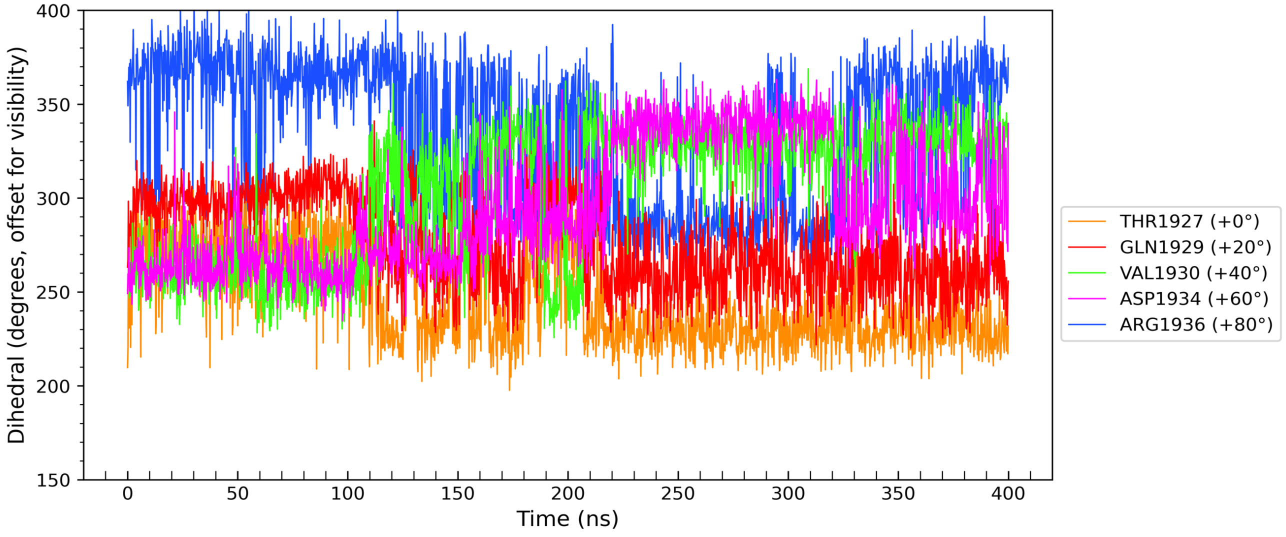

Supplemental Figure 4. Raw φ dihedral data for residues at each pivot region, computed from the MD trajectory initialized with random seed 1. The dihedrals correspond to residues identified in the clusters shown in Figure 1B. **(A)** DDB1–CUL4A pivot region. **(B)** DDB1–CRBN interface. **(C)** CRBN–BRD4 interface. **(D)** CUL4A C-terminal region. **(E)** RBX1–UB interface.

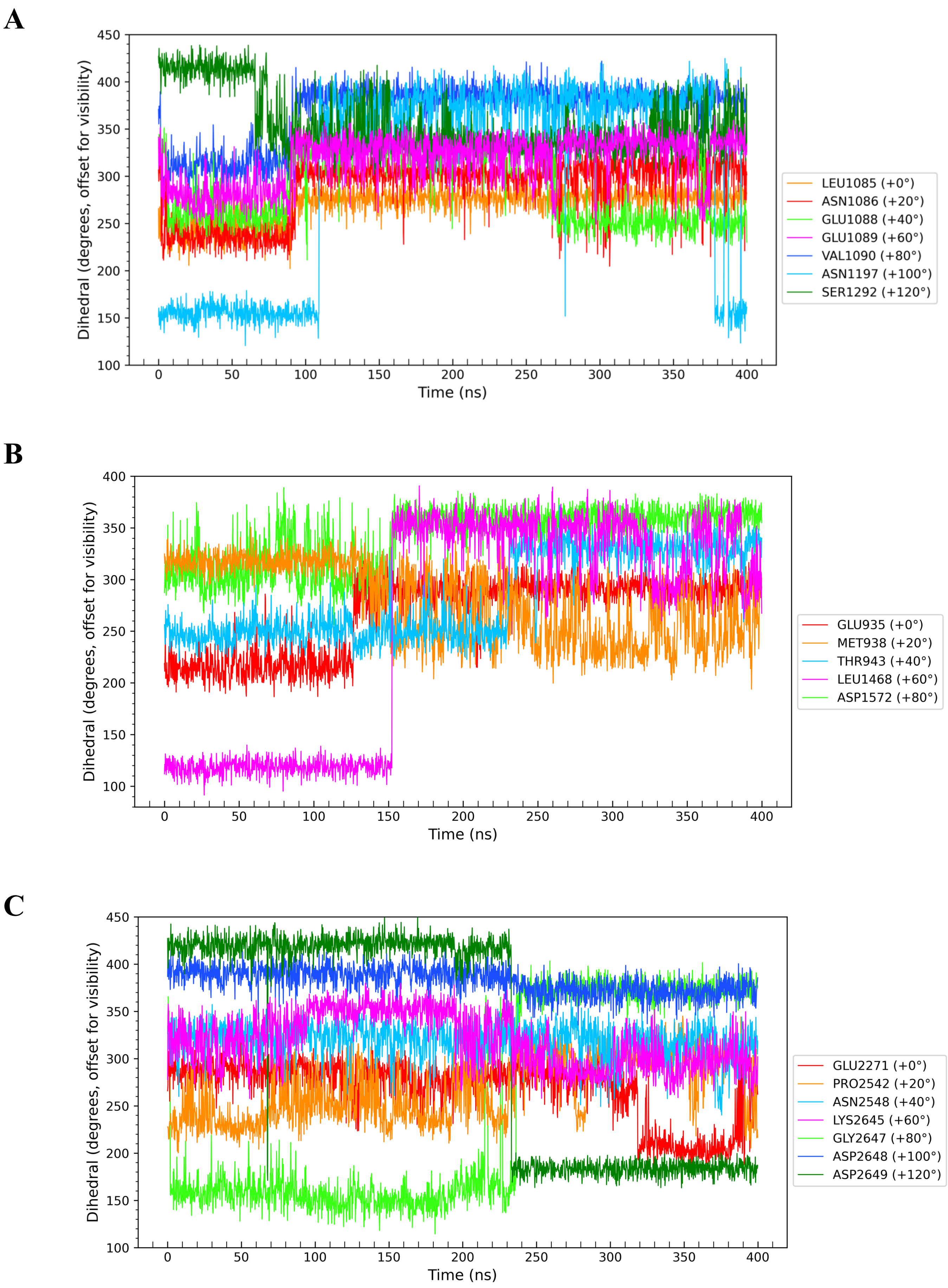
A
Dihedral (degrees, offset for visibility)
Time (ns)
LEU1085 (+0°)
ASN1086 (+20°)
GLU1088 (+40°)
GLU1089 (+60°)
VAL1090 (+80°)
ASN1197 (+100°)
SER1292 (+120°)
B
Dihedral (degrees, offset for visibility)
Time (ns)
GLU935 (+0°)
MET938 (+20°)
THR943 (+40°)
LEU1468 (+60°)
ASP1572 (+80°)
C
Dihedral (degrees, offset for visibility)
Time (ns)
GLU2271 (+0°)
PRO2542 (+20°)
ASN2548 (+40°)
LYS2645 (+60°)
GLY2647 (+80°)
ASP2648 (+100°)
ASP2649 (+120°)

D

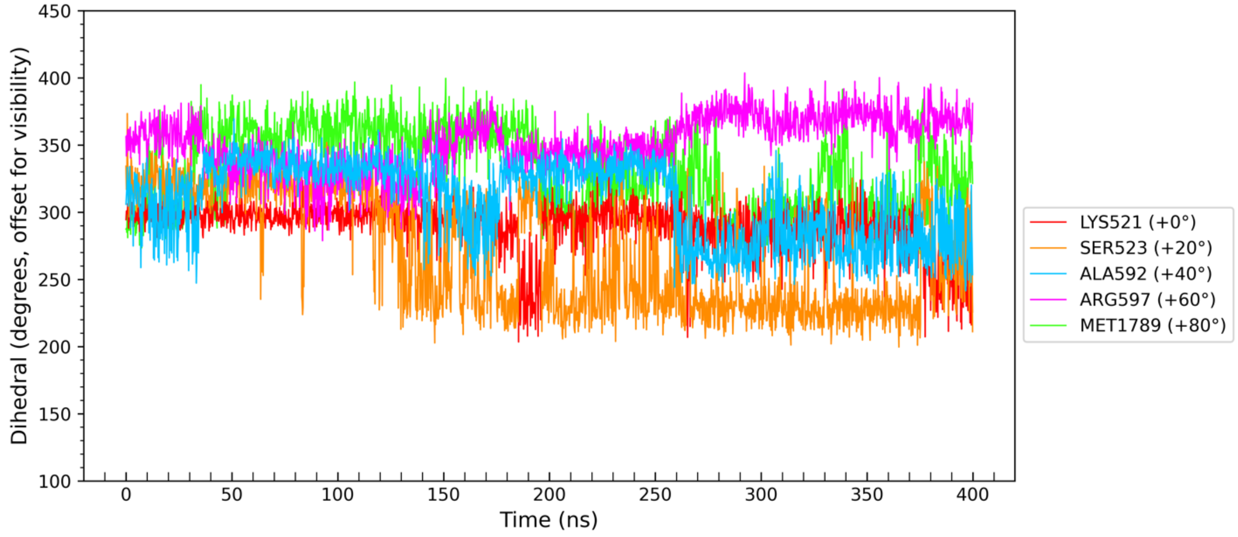

E

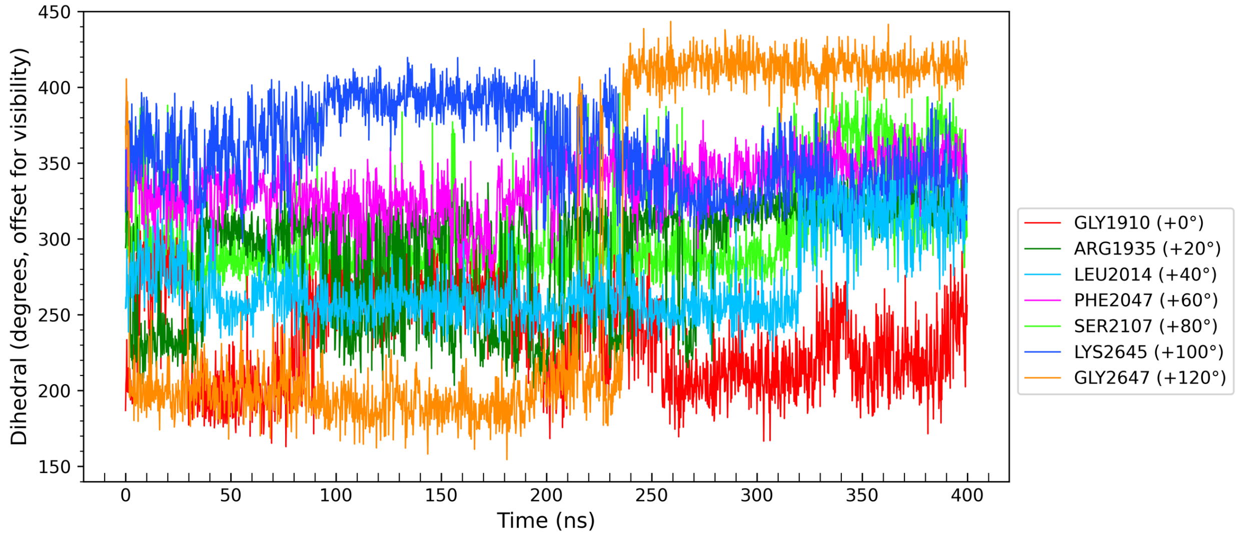

Supplemental Figure 5. Raw φ dihedral data for residues at each pivot region, computed from the MD trajectory initialized with random seed 2. The dihedrals correspond to residues identified in the clusters shown in SI Figure 3A. **(A)** DDB1–CUL4A pivot region. **(B)** DDB1–CRBN interface. **(C)** CRBN–BRD4 interface. **(D)** CUL4A C-terminal region. **(E)** RBX1–UB interface.

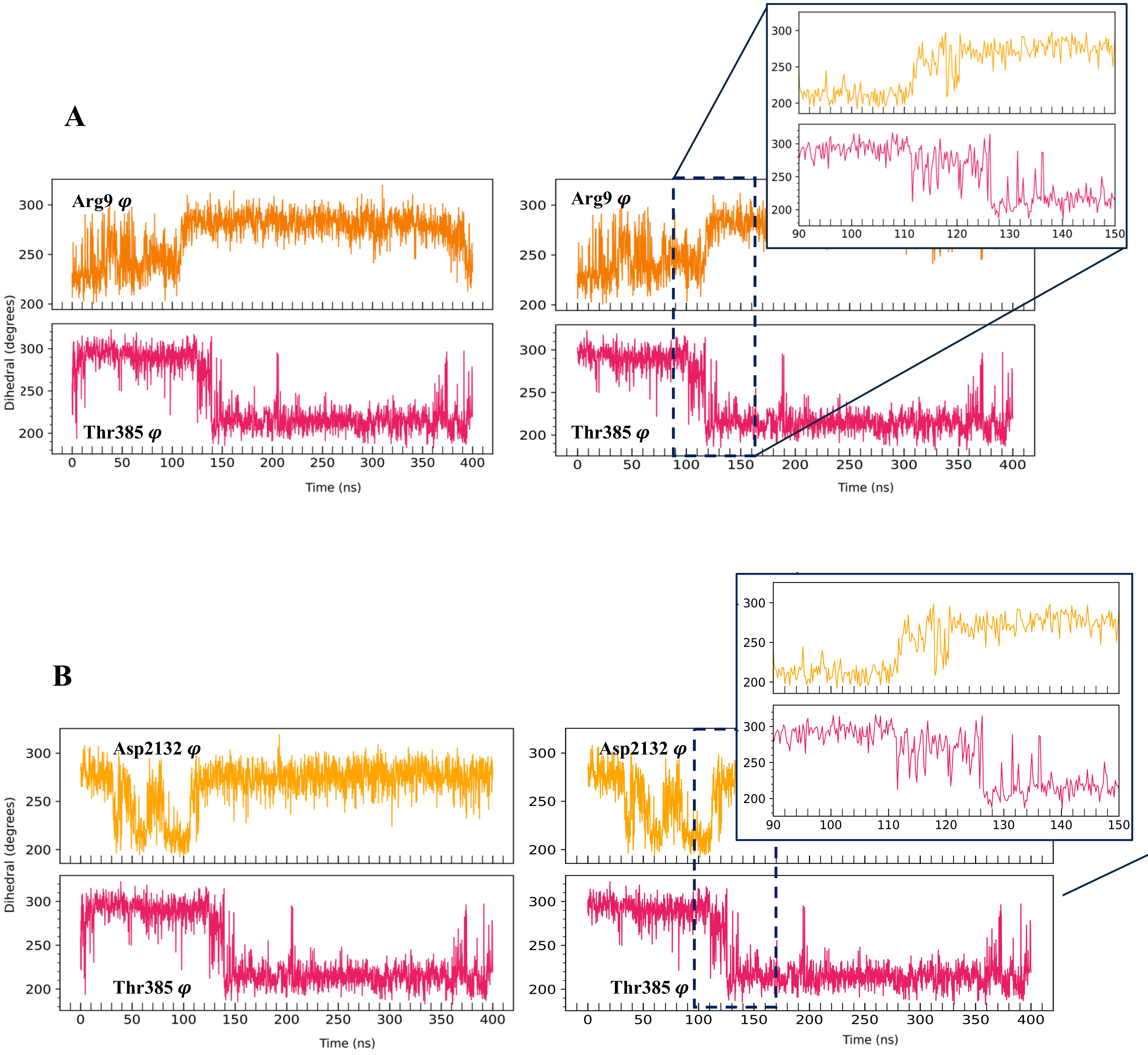

Supplemental Figure 6. Dihedral φ time series illustrating lag-dependent alignment of fluctuations, computed from the MD trajectory initialized with random seed 1. **(A) Left:** Arg9 (orange) and Thr385 (pink) dihedrals shown at zero lag (L = 0). **Right:** Thr385 dihedral shifted by ~30 ns (L = 30). **(B) Left:** Asp2132 (orange) and Thr385 (pink) dihedrals at zero lag (L = 0). **Right:** Thr385 dihedral shifted by 30 ns (L = 30). In both (A) and (B), dark blue dashed lines indicate regions of aligned fluctuations, highlighted in the zoomed panels.

A

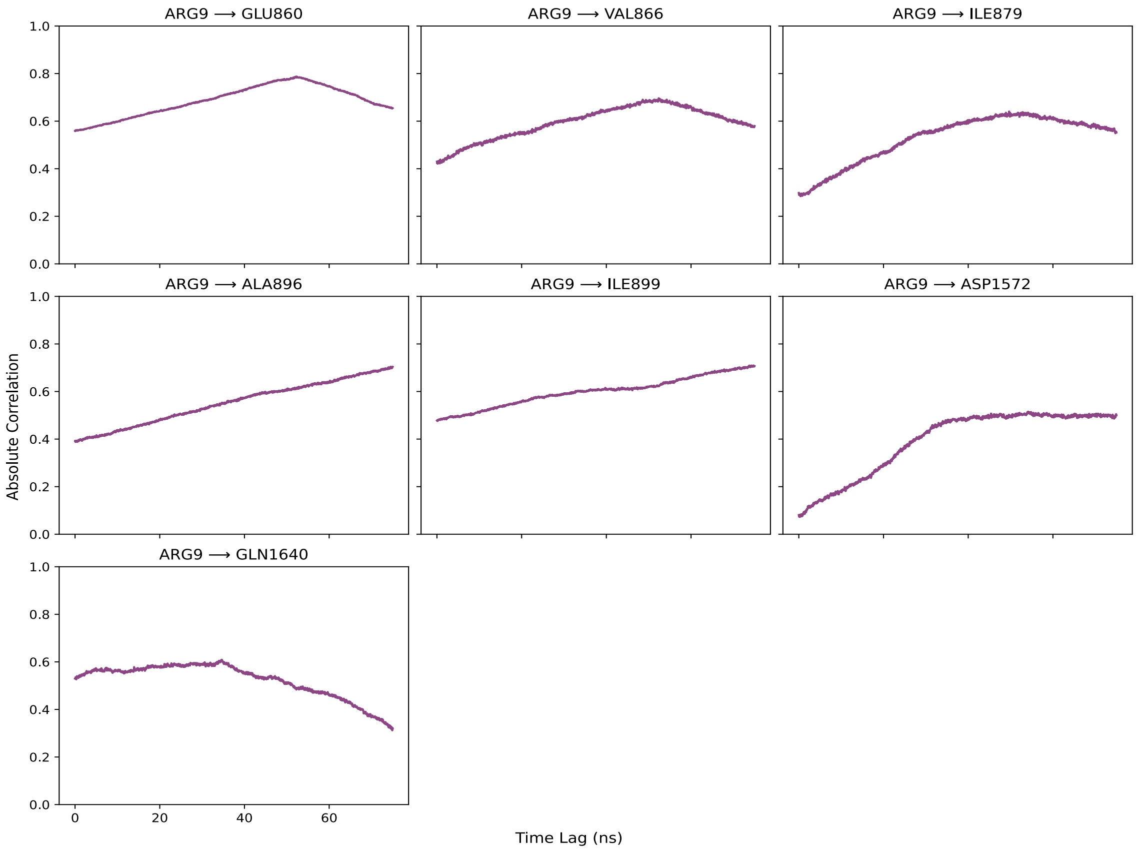
ARG9 ⟶ GLU860
ARG9 ⟶ VAL866
ARG9 ⟶ ILE879
ARG9 ⟶ ALA896
ARG9 ⟶ ILE899
ARG9 ⟶ ASP1572
ARG9 ⟶ GLN1640
Absolute Correlation
1.0
0.8
0.6
0.4
0.2
0.0
0
20
40
60
Time Lag (ns)

B

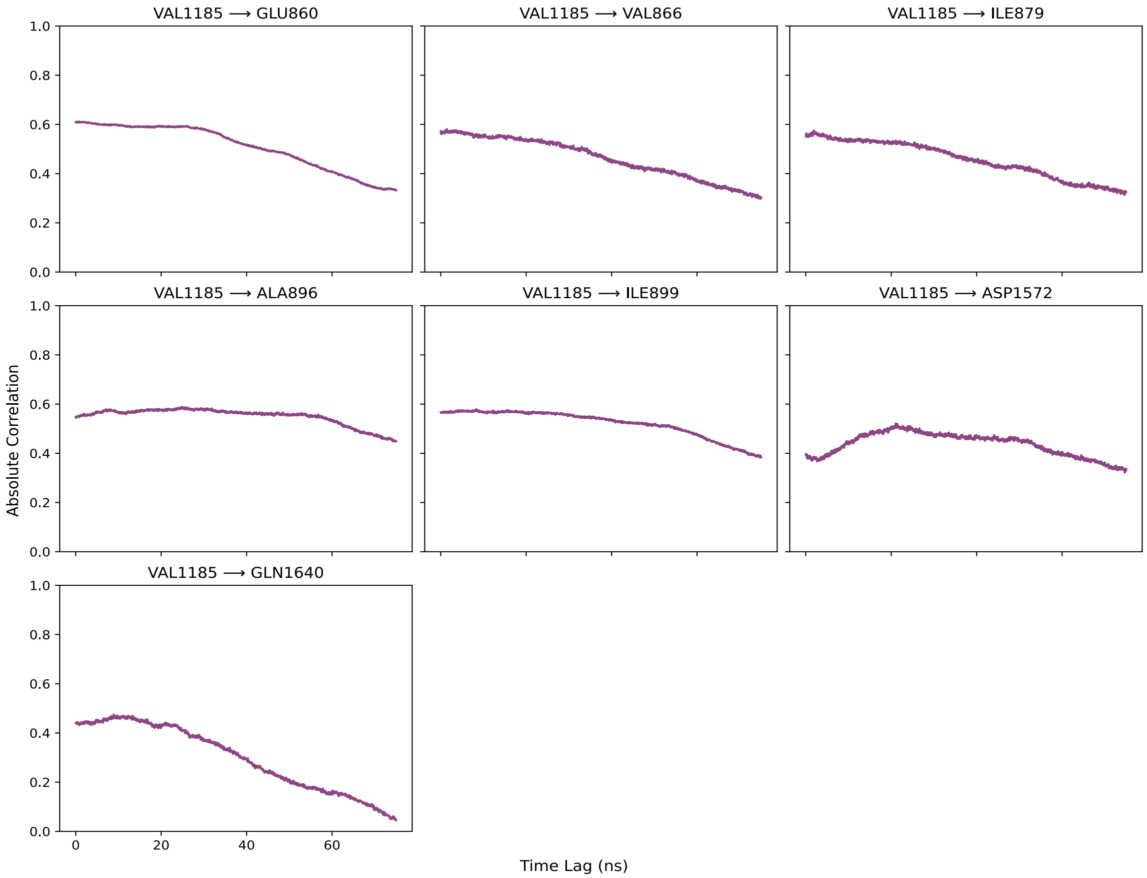
VAL1185 ⟶ GLU860
VAL1185 ⟶ VAL866
VAL1185 ⟶ ILE879
VAL1185 ⟶ ALA896
VAL1185 ⟶ ILE899
VAL1185 ⟶ ASP1572
VAL1185 ⟶ GLN1640
Absolute Correlation
1.0
0.8
0.6
0.4
0.2
0.0
0
20
40
60
Time Lag (ns)

C

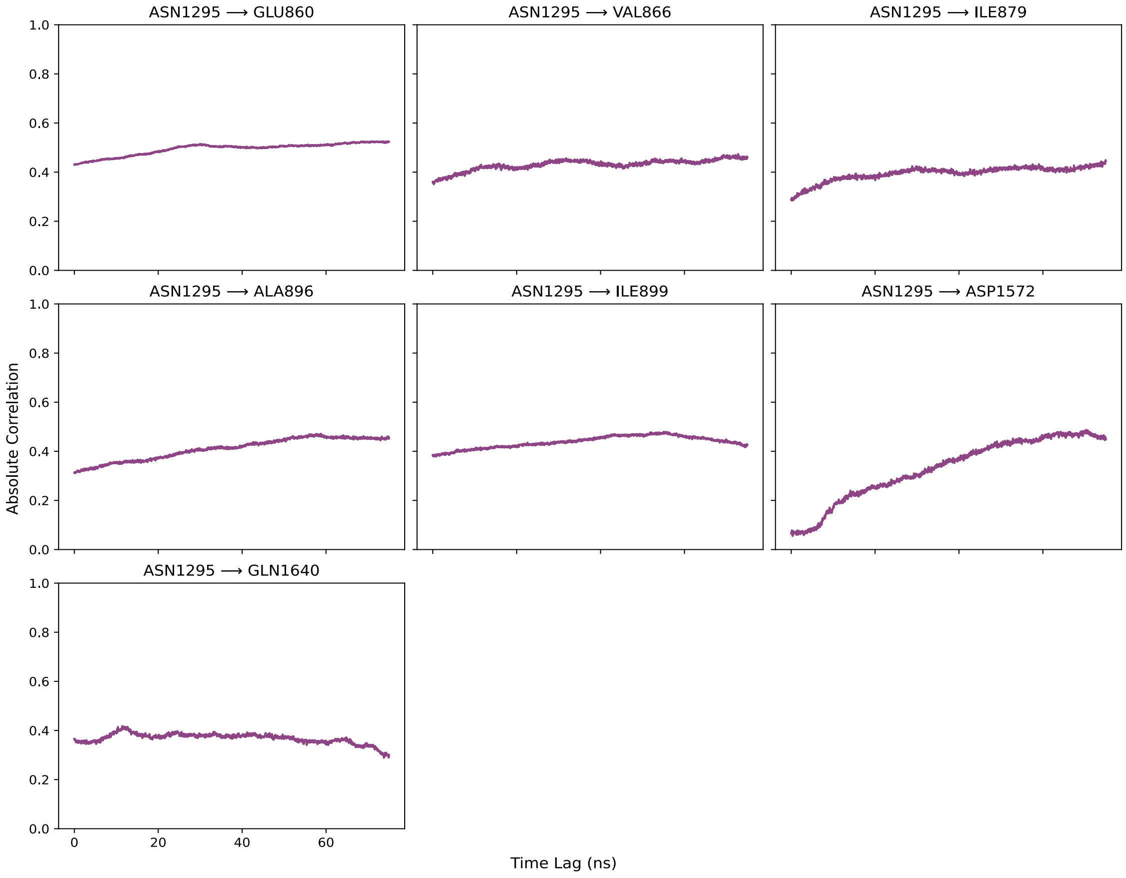
ASN1295 ⟶ GLU860
ASN1295 ⟶ VAL866
ASN1295 ⟶ ILE879
ASN1295 ⟶ ALA896
ASN1295 ⟶ ILE899
ASN1295 ⟶ ASP1572
ASN1295 ⟶ GLN1640
Absolute Correlation
Time Lag (ns)
0.0
0.2
0.4
0.6
0.8
1.0
0
20
40
60

D

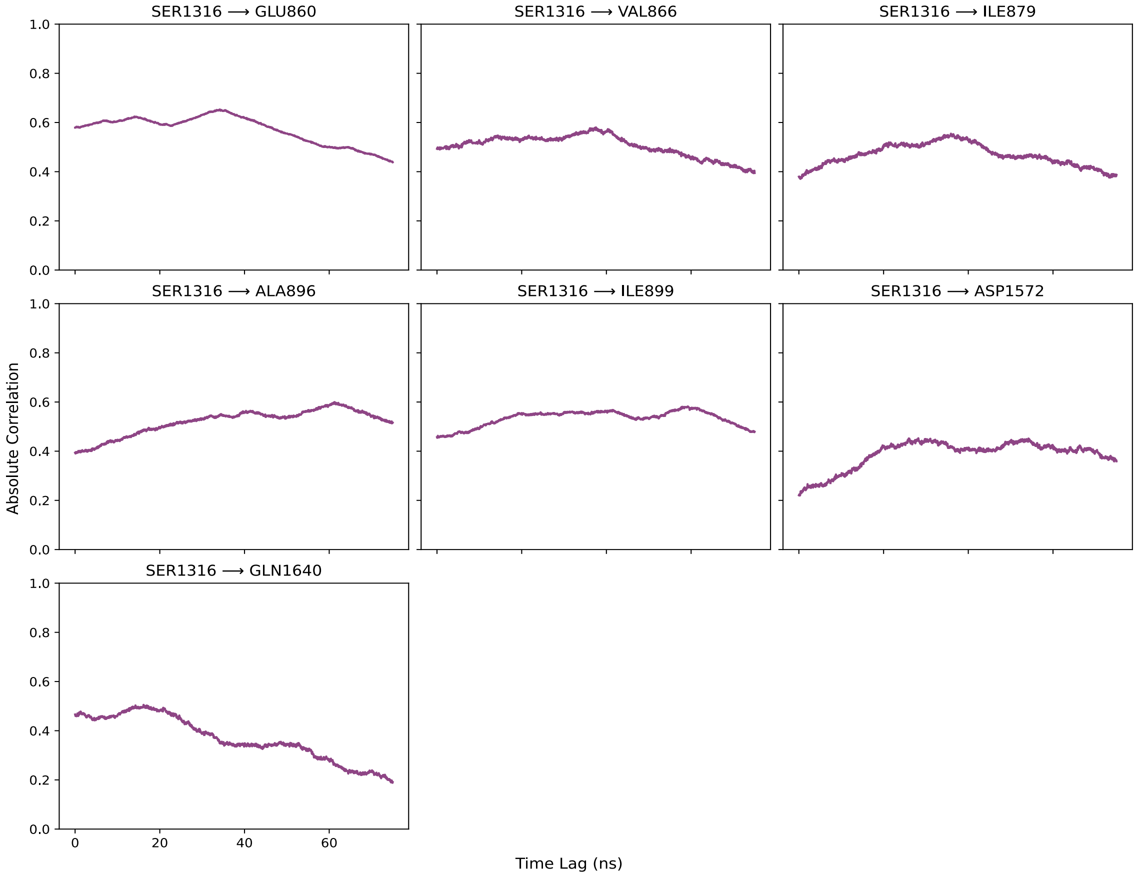
SER1316 ⟶ GLU860
SER1316 ⟶ VAL866
SER1316 ⟶ ILE879
SER1316 ⟶ ALA896
SER1316 ⟶ ILE899
SER1316 ⟶ ASP1572
SER1316 ⟶ GLN1640
Absolute Correlation
Time Lag (ns)
0.0
0.2
0.4
0.6
0.8
1.0
0
20
40
60

E

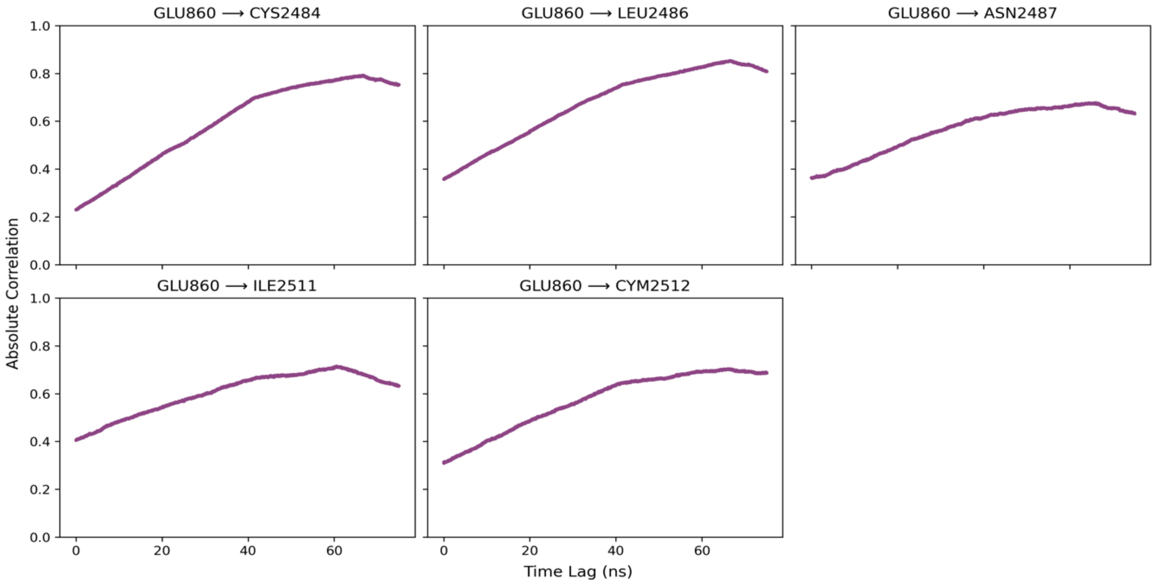
GLU860 ⟶ CYS2484
GLU860 ⟶ LEU2486
GLU860 ⟶ ASN2487
GLU860 ⟶ ILE2511
GLU860 ⟶ CYM2512
Absolute Correlation
Time Lag (ns)

F

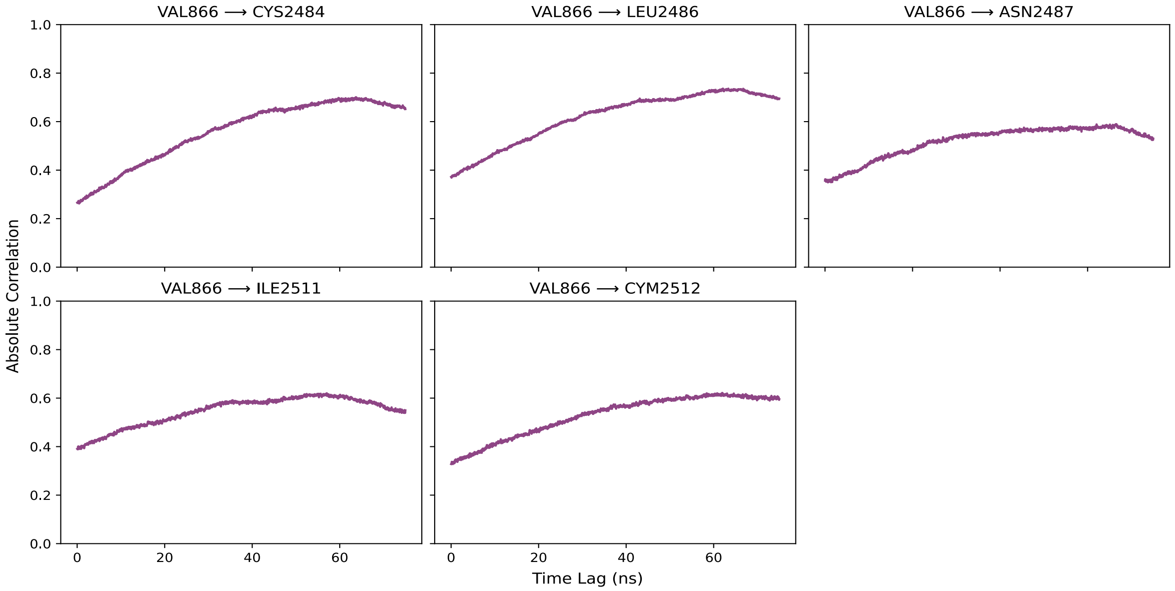
VAL866 ⟶ CYS2484
VAL866 ⟶ LEU2486
VAL866 ⟶ ASN2487
VAL866 ⟶ ILE2511
VAL866 ⟶ CYM2512
Absolute Correlation
Time Lag (ns)

G

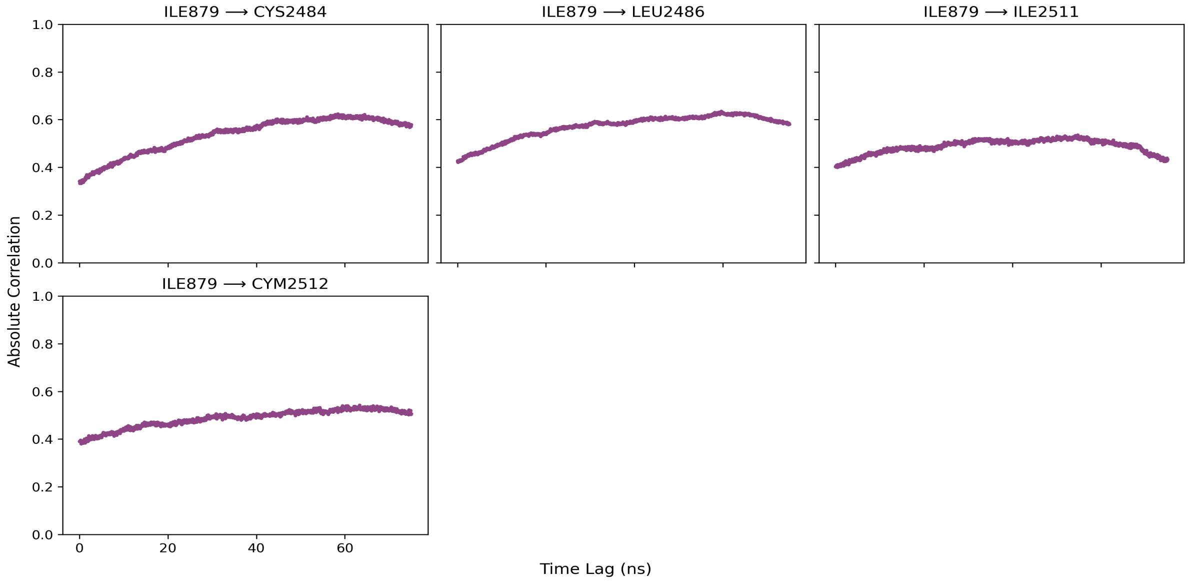
ILE879 ⟶ CYS2484
ILE879 ⟶ LEU2486
ILE879 ⟶ ILE2511
ILE879 ⟶ CYM2512
Absolute Correlation
Time Lag (ns)

H

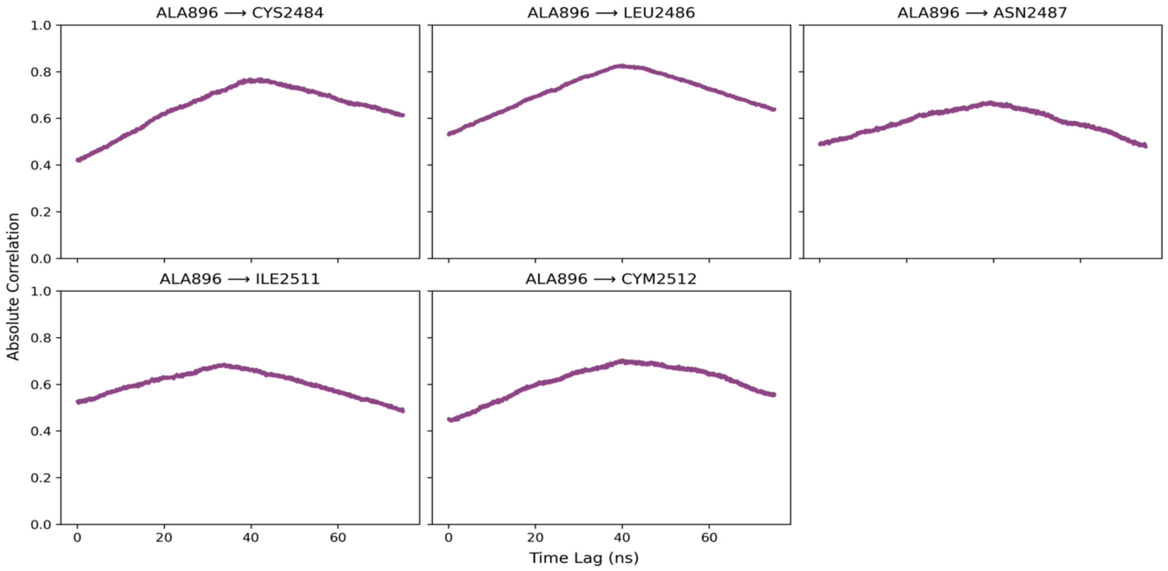
ALA896 ⟶ CYS2484
ALA896 ⟶ LEU2486
ALA896 ⟶ ASN2487
ALA896 ⟶ ILE2511
ALA896 ⟶ CYM2512
Absolute Correlation
Time Lag (ns)

I

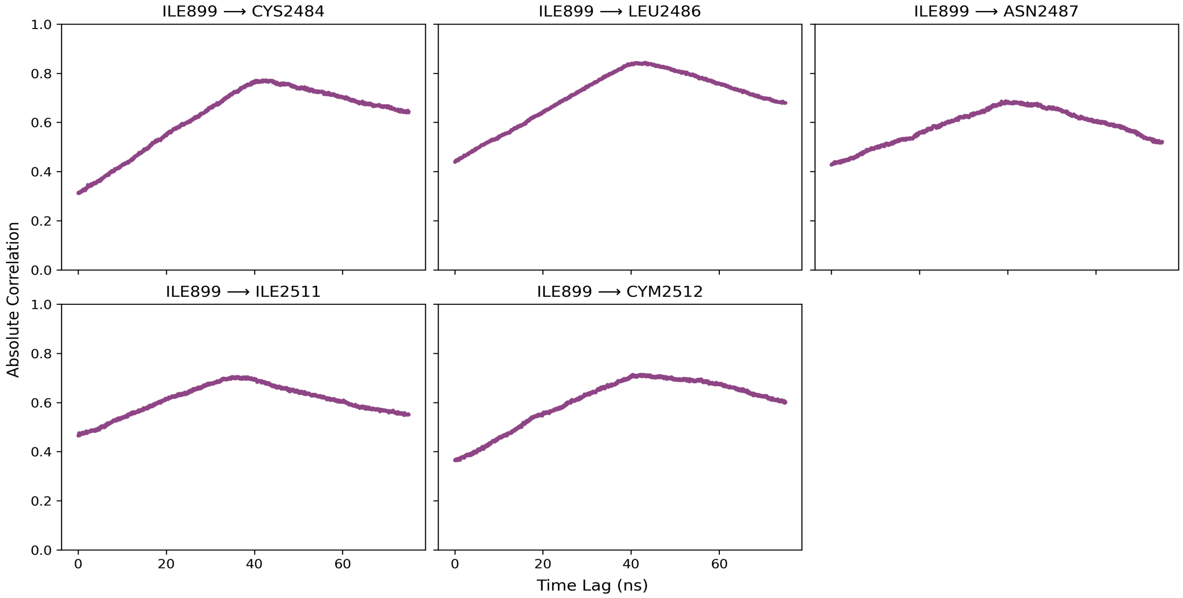
ILE899 ⟶ CYS2484
ILE899 ⟶ LEU2486
ILE899 ⟶ ASN2487
ILE899 ⟶ ILE2511
ILE899 ⟶ CYM2512
Absolute Correlation
Time Lag (ns)

J

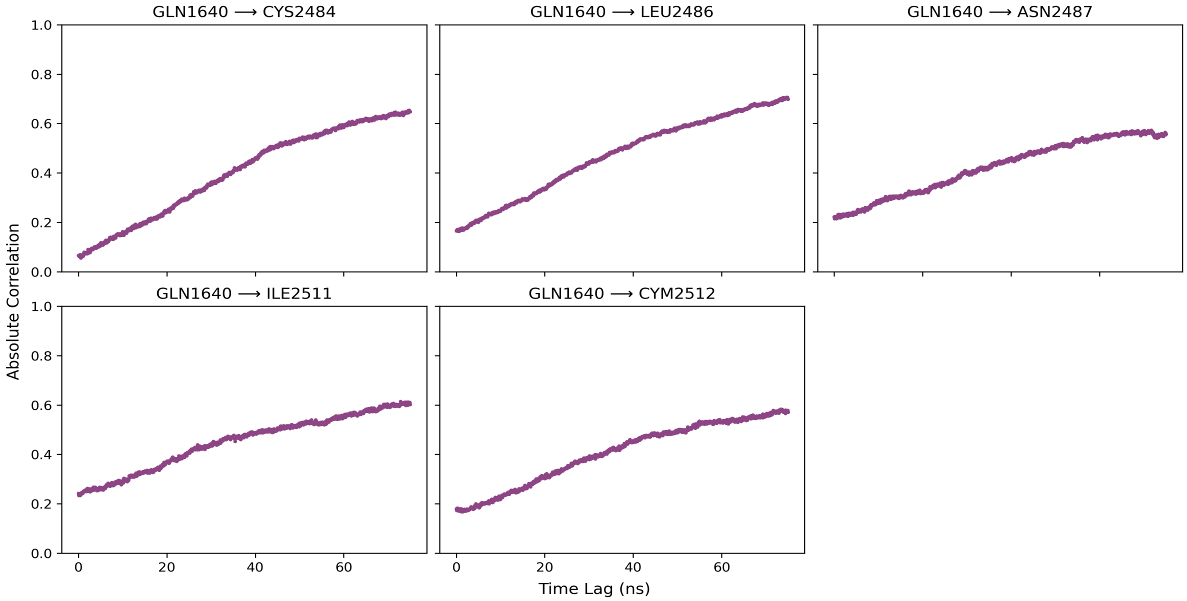
GLN1640 ⟶ CYS2484
GLN1640 ⟶ LEU2486
GLN1640 ⟶ ASN2487
GLN1640 ⟶ ILE2511
GLN1640 ⟶ CYM2512
Absolute Correlation
Time Lag (ns)

K

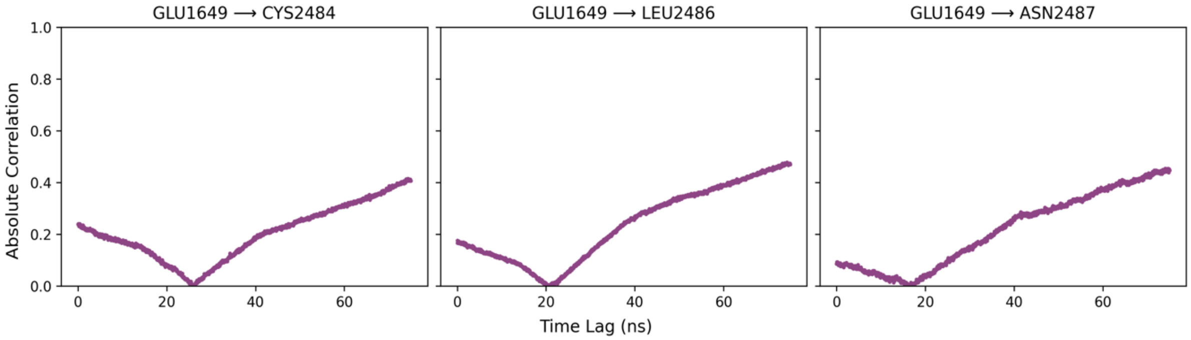

Supplemental Figure 7. Time-dependent torsional correlation plots for the propagation pathways within the substrate recognition module, computed from the MD trajectory initialized with random seed 1. Arrows indicate direction of propagation. **(A)** Correlations of Arg9 with DDB1-CRBN interface residues. **(B)** Val1185 with DDB1-CRBN interface residues. **(C)** Asn1295 with DDB1-CRBN interface residues. **(D)** Ser1316 with DDB1-CRBN interface residues. **(E)** Glu860 with CRBN-BRD4 interface residues. **(F)** Val866 with CRBN—BRD4 interface residues. **(G)** Ile879 with CRBN-BRD4 interface residues. **(H)** Ala896 with CRBN-BRD4 interface residues. **(I)** Ile899 with CRBN-BRD4 interface residues. **(J)** Gln1640 with CRBN-BRD4 interface residues. **(K)** Glu1649 with CRBN-BRD4 interface residues.

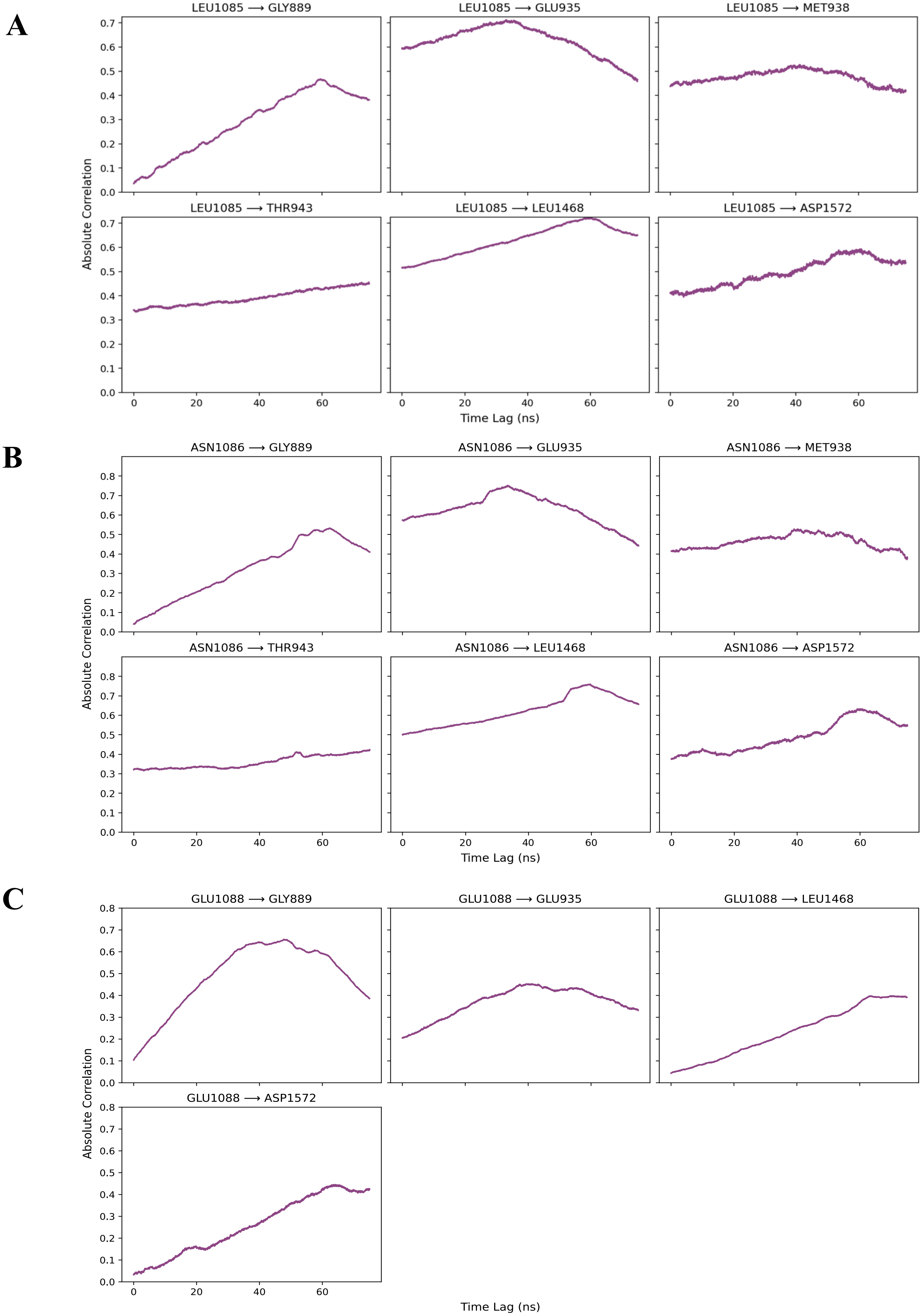
A
LEU1085 ⟶ GLY889
LEU1085 ⟶ GLU935
LEU1085 ⟶ MET938
LEU1085 ⟶ THR943
LEU1085 ⟶ LEU1468
LEU1085 ⟶ ASP1572
Absolute Correlation
Time Lag (ns)
B
ASN1086 ⟶ GLY889
ASN1086 ⟶ GLU935
ASN1086 ⟶ MET938
ASN1086 ⟶ THR943
ASN1086 ⟶ LEU1468
ASN1086 ⟶ ASP1572
Absolute Correlation
Time Lag (ns)
C
GLU1088 ⟶ GLY889
GLU1088 ⟶ GLU935
GLU1088 ⟶ LEU1468
GLU1088 ⟶ ASP1572
Absolute Correlation
Time Lag (ns)

D

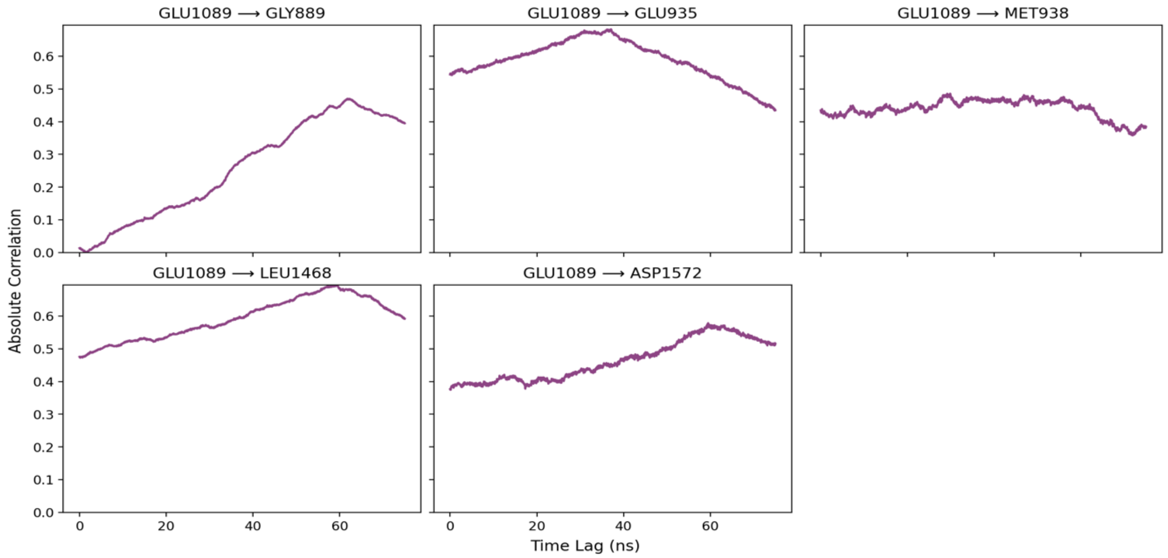
GLU1089 ⟶ GLY889
GLU1089 ⟶ GLU935
GLU1089 ⟶ MET938
GLU1089 ⟶ LEU1468
GLU1089 ⟶ ASP1572
Absolute Correlation
Time Lag (ns)

E

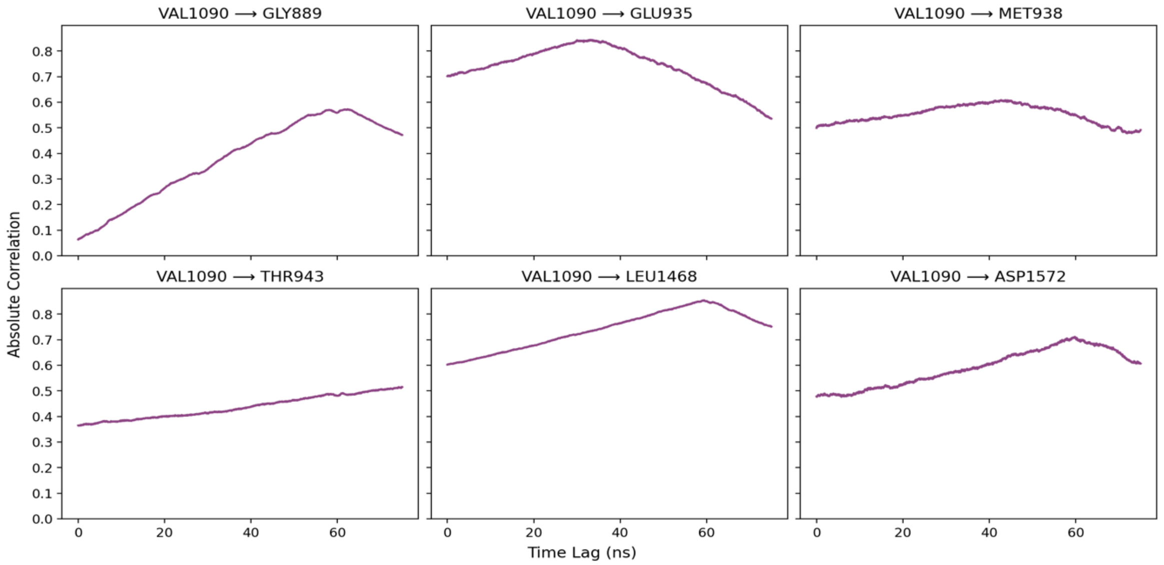
VAL1090 ⟶ GLY889
VAL1090 ⟶ GLU935
VAL1090 ⟶ MET938
VAL1090 ⟶ THR943
VAL1090 ⟶ LEU1468
VAL1090 ⟶ ASP1572
Absolute Correlation
Time Lag (ns)

F

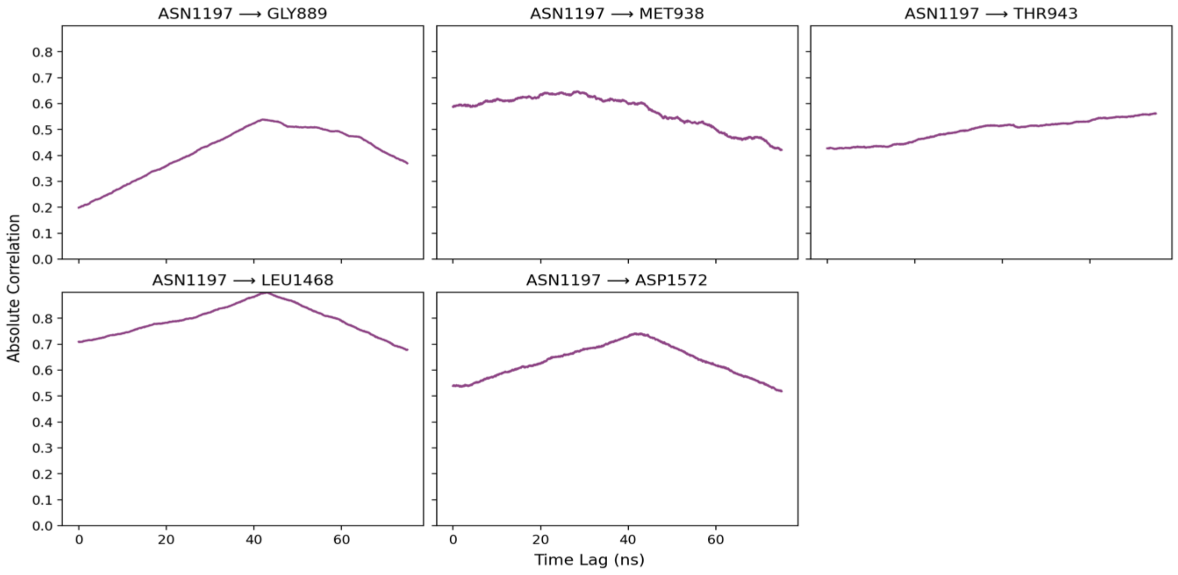
ASN1197 ⟶ GLY889
ASN1197 ⟶ MET938
ASN1197 ⟶ THR943
ASN1197 ⟶ LEU1468
ASN1197 ⟶ ASP1572
Absolute Correlation
Time Lag (ns)

G

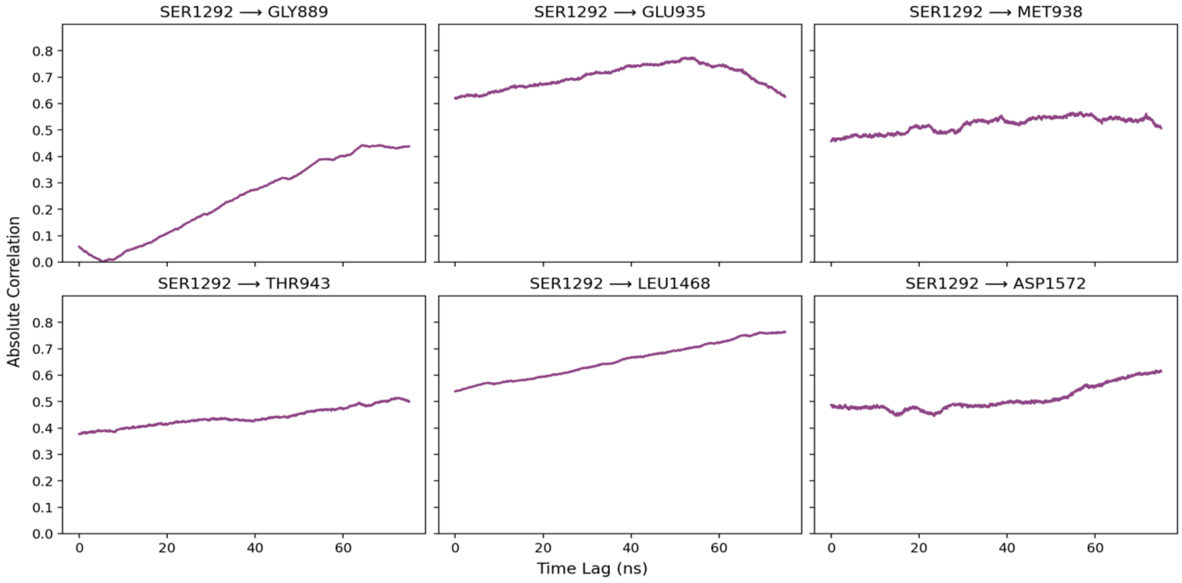
SER1292 ⟶ GLY889
SER1292 ⟶ GLU935
SER1292 ⟶ MET938
SER1292 ⟶ THR943
SER1292 ⟶ LEU1468
SER1292 ⟶ ASP1572
Absolute Correlation
Time Lag (ns)

H

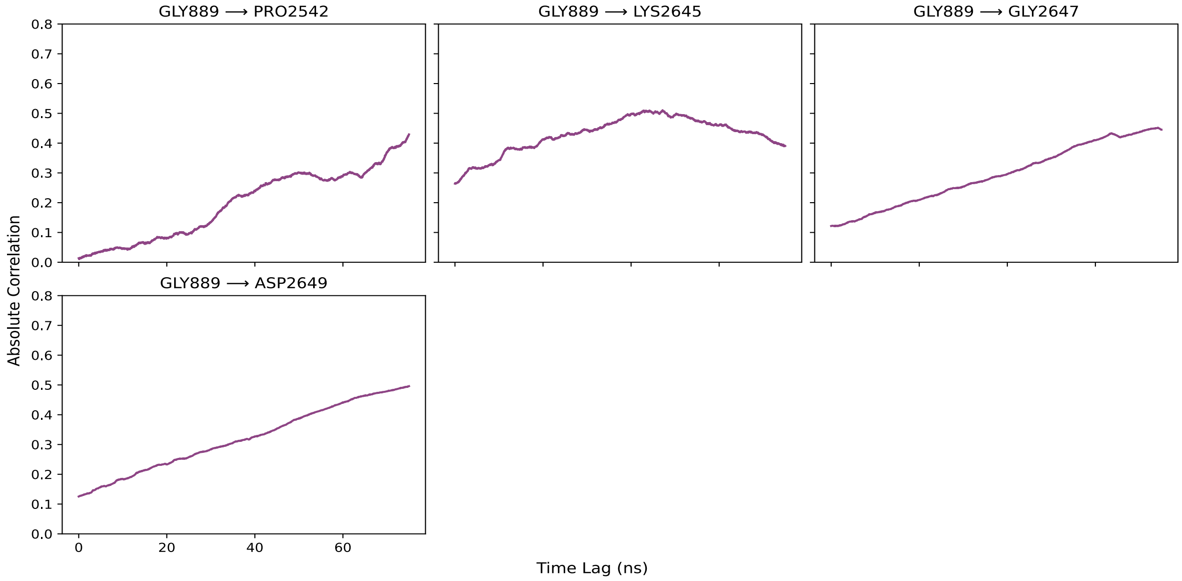
GLY889 ⟶ PRO2542
GLY889 ⟶ LYS2645
GLY889 ⟶ GLY2647
GLY889 ⟶ ASP2649
Absolute Correlation
Time Lag (ns)

I

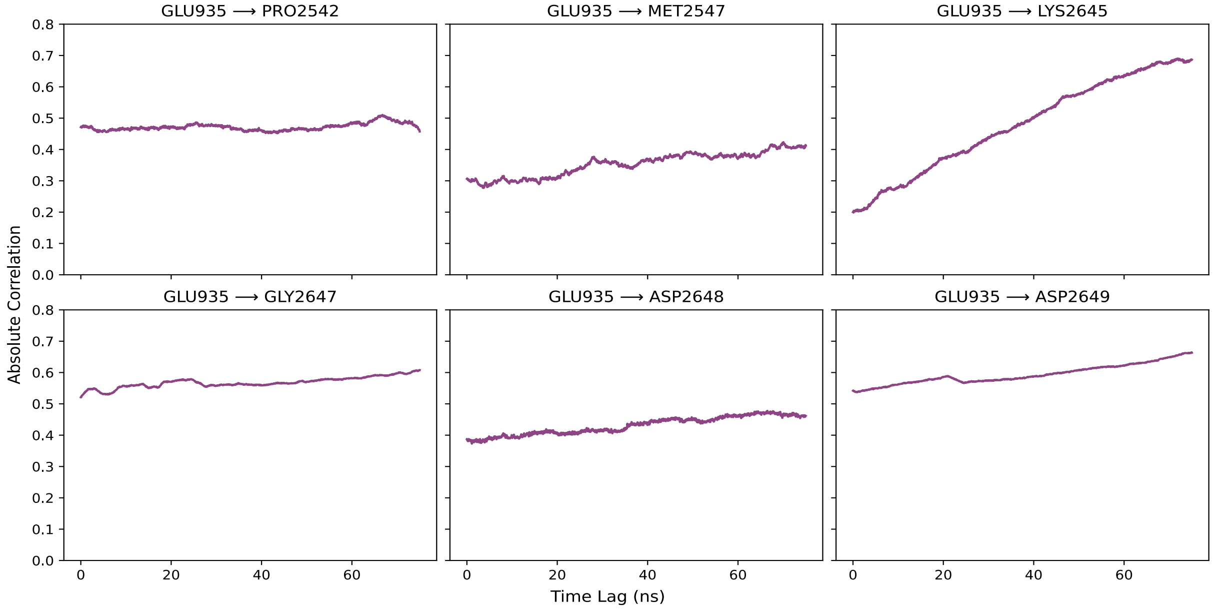
GLU935 ⟶ PRO2542
GLU935 ⟶ MET2547
GLU935 ⟶ LYS2645
GLU935 ⟶ GLY2647
GLU935 ⟶ ASP2648
GLU935 ⟶ ASP2649
Absolute Correlation
Time Lag (ns)

J

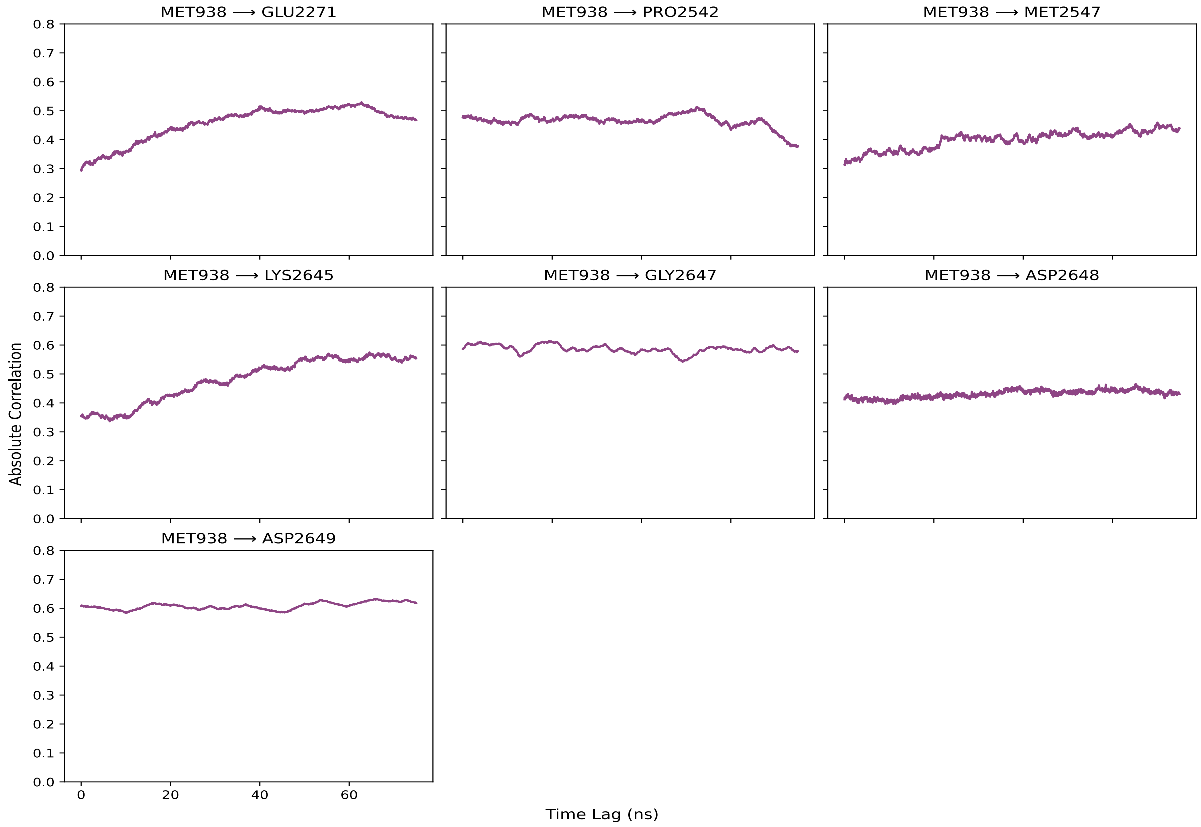

MET938 ⟶ GLU2271
MET938 ⟶ PRO2542
MET938 ⟶ MET2547
MET938 ⟶ LYS2645
MET938 ⟶ GLY2647
MET938 ⟶ ASP2648
MET938 ⟶ ASP2649
Absolute Correlation
0.0 0.1 0.2 0.3 0.4 0.5 0.6 0.7 0.8
0 20 40 60
Time Lag (ns)

K

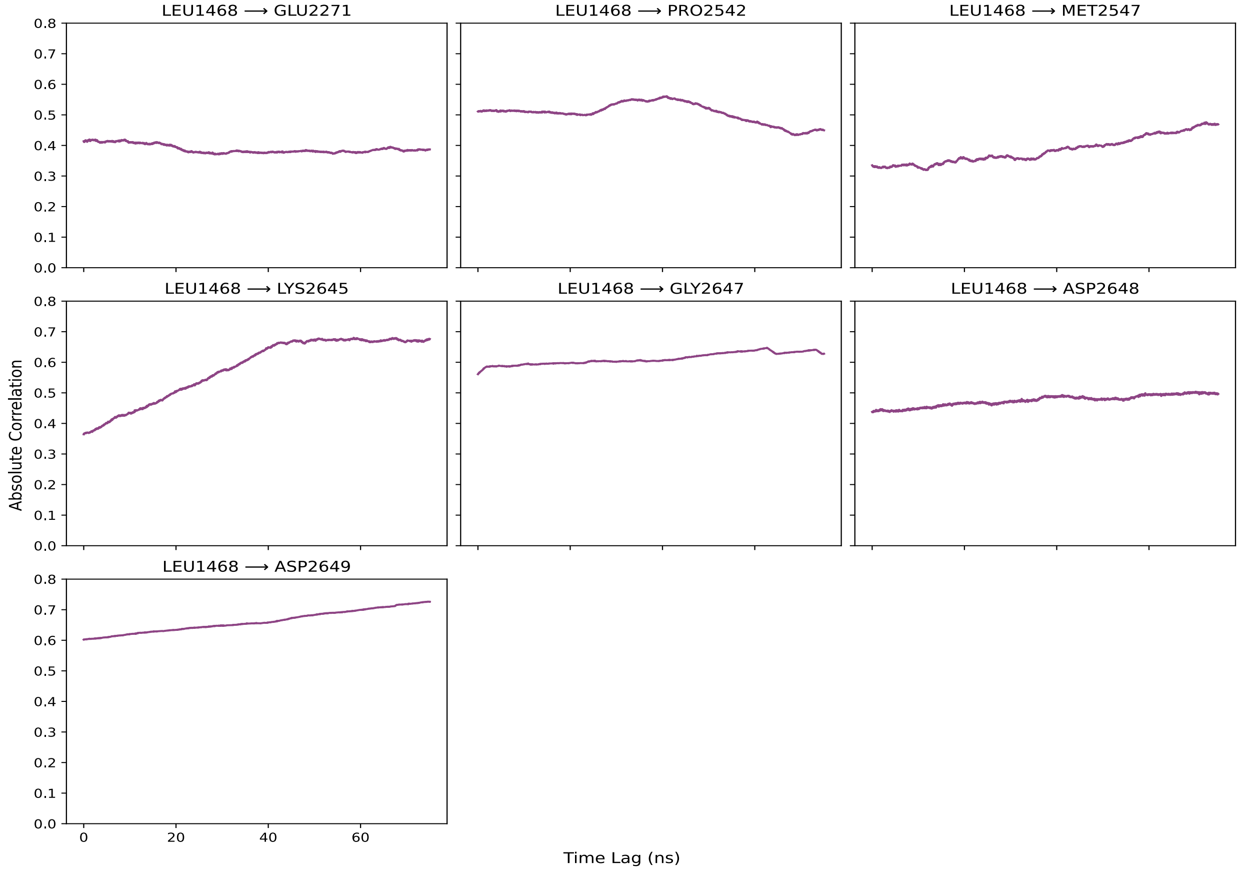

LEU1468 ⟶ GLU2271
LEU1468 ⟶ PRO2542
LEU1468 ⟶ MET2547
LEU1468 ⟶ LYS2645
LEU1468 ⟶ GLY2647
LEU1468 ⟶ ASP2648
LEU1468 ⟶ ASP2649
Absolute Correlation
0.0 0.1 0.2 0.3 0.4 0.5 0.6 0.7 0.8
0 20 40 60
Time Lag (ns)

L

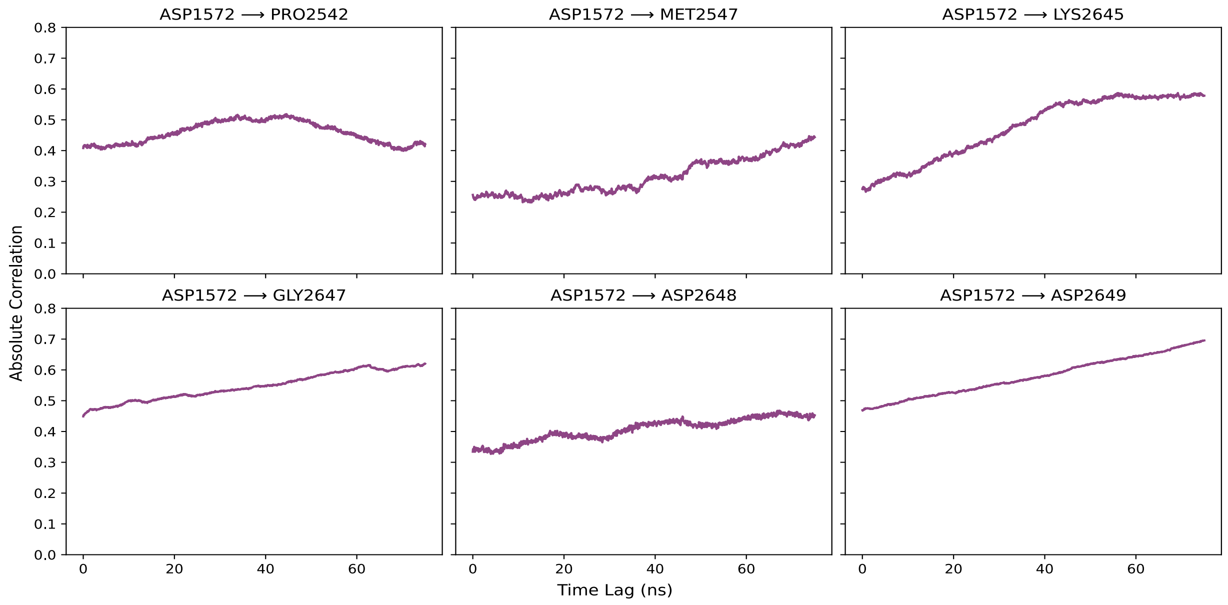

Supplemental Figure 8. Time-dependent torsional correlation plots for propagation paths within the substrate recognition module, computed from the MD trajectory initialized with random seed 2. Arrows indicate direction of propagation. **(A)** Correlations of Leu1085 with DDB1-CRBN interface residues. **(B)** Asn1086 with DDB1-CRBN interface residues. **(C)** Glu1088 with DDB1-CRBN interface residues. (**D)** Glu1089 with DDB1-CRBN interface residues. **(E)** Val1090 with DDB1-CRBN interface residues. **(F)** Asn1197 with DDB1-CRBN interface residues. **(G)** Ser1292 with DDB1-CRBN interface residues. **(H)** Gly889 with CRBN-BRD4 interface residues. **(I)** Glu935 with CRBN-BRD4 interface residues. **(J)** Met938 with CRBN-BRD4 interface residues. **(K)** Leu1468 with CRBN-BRD4 interface residues. **(L)** Asp1572 with CRBN-BRD4 interface residues. Comparable time-lagged correlations patterns are observed across both independently seeded trajectories.

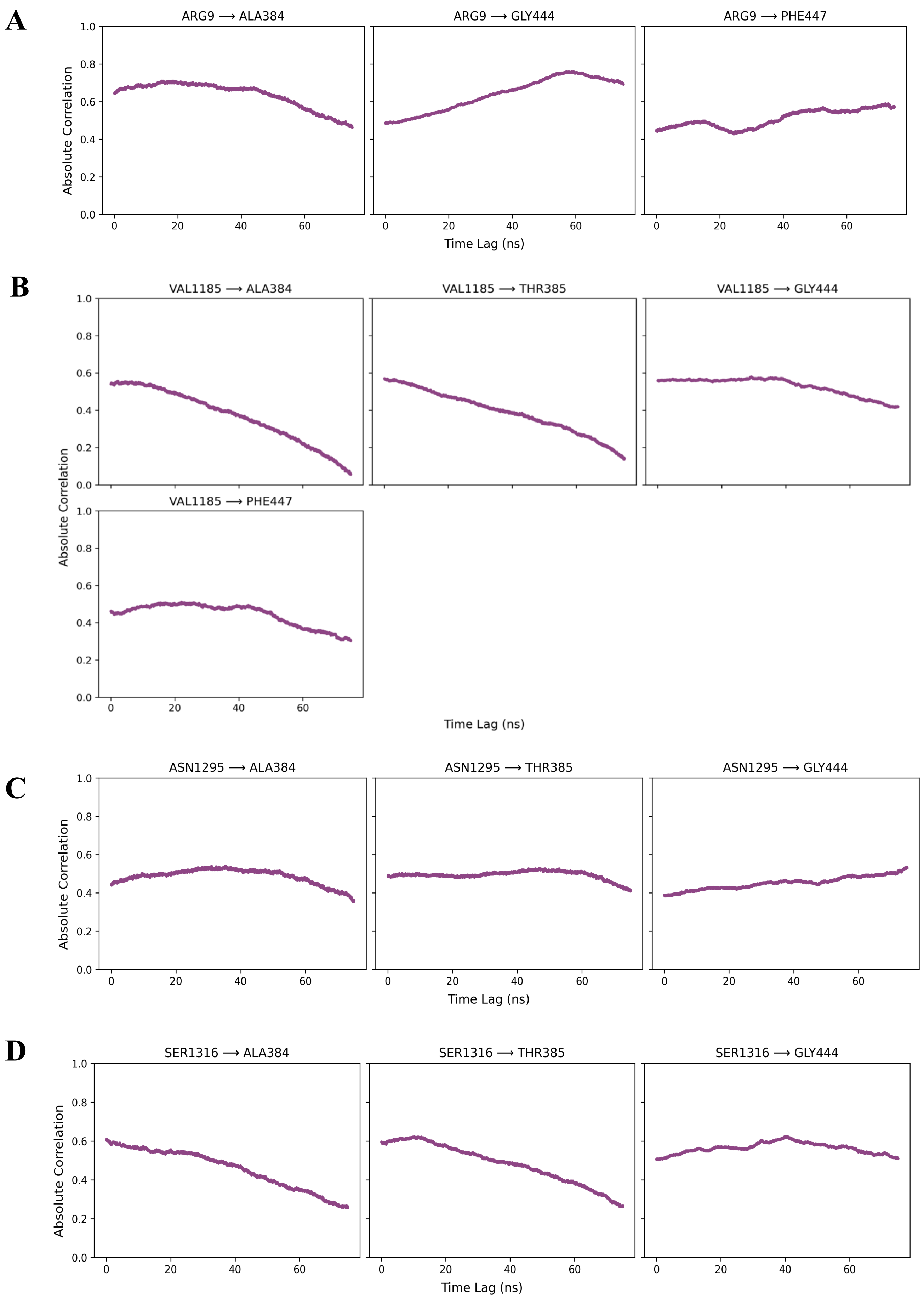
A
ARG9 ⟶ ALA384
ARG9 ⟶ GLY444
ARG9 ⟶ PHE447
Absolute Correlation
Time Lag (ns)
B
VAL1185 ⟶ ALA384
VAL1185 ⟶ THR385
VAL1185 ⟶ GLY444
VAL1185 ⟶ PHE447
Absolute Correlation
Time Lag (ns)
C
ASN1295 ⟶ ALA384
ASN1295 ⟶ THR385
ASN1295 ⟶ GLY444
Absolute Correlation
Time Lag (ns)
D
SER1316 ⟶ ALA384
SER1316 ⟶ THR385
SER1316 ⟶ GLY444
Absolute Correlation
Time Lag (ns)

E

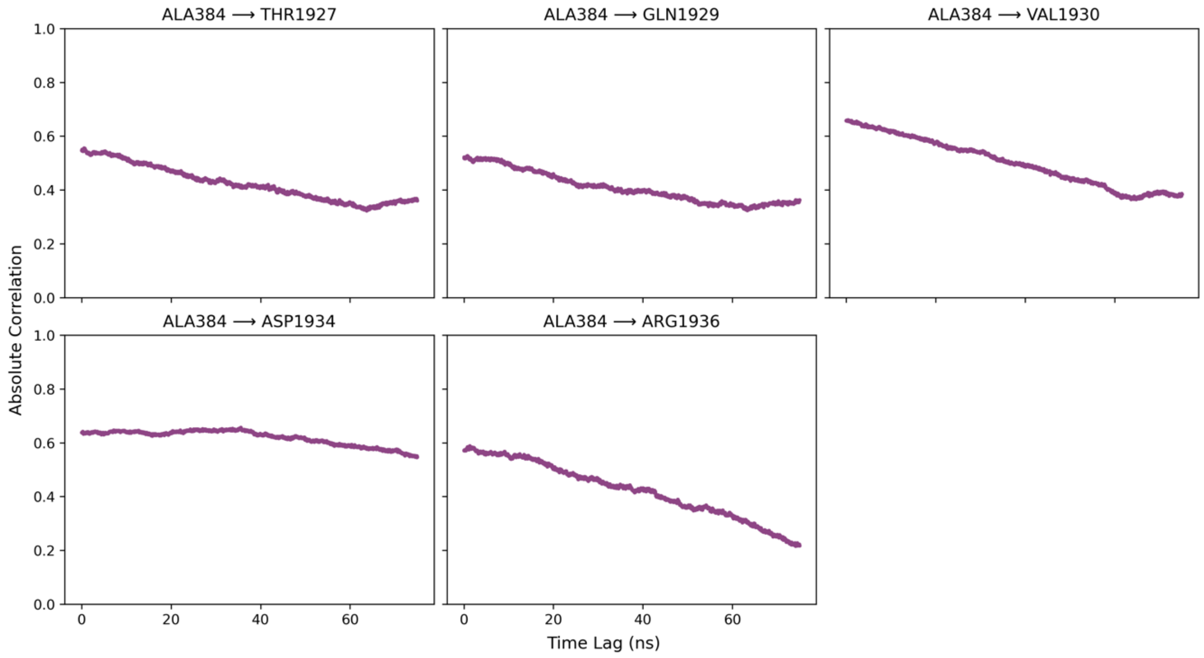
ALA384 ⟶ THR1927
ALA384 ⟶ GLN1929
ALA384 ⟶ VAL1930
ALA384 ⟶ ASP1934
ALA384 ⟶ ARG1936
Absolute Correlation
Time Lag (ns)
0.0
0.2
0.4
0.6
0.8
1.0
0
20
40
60

F

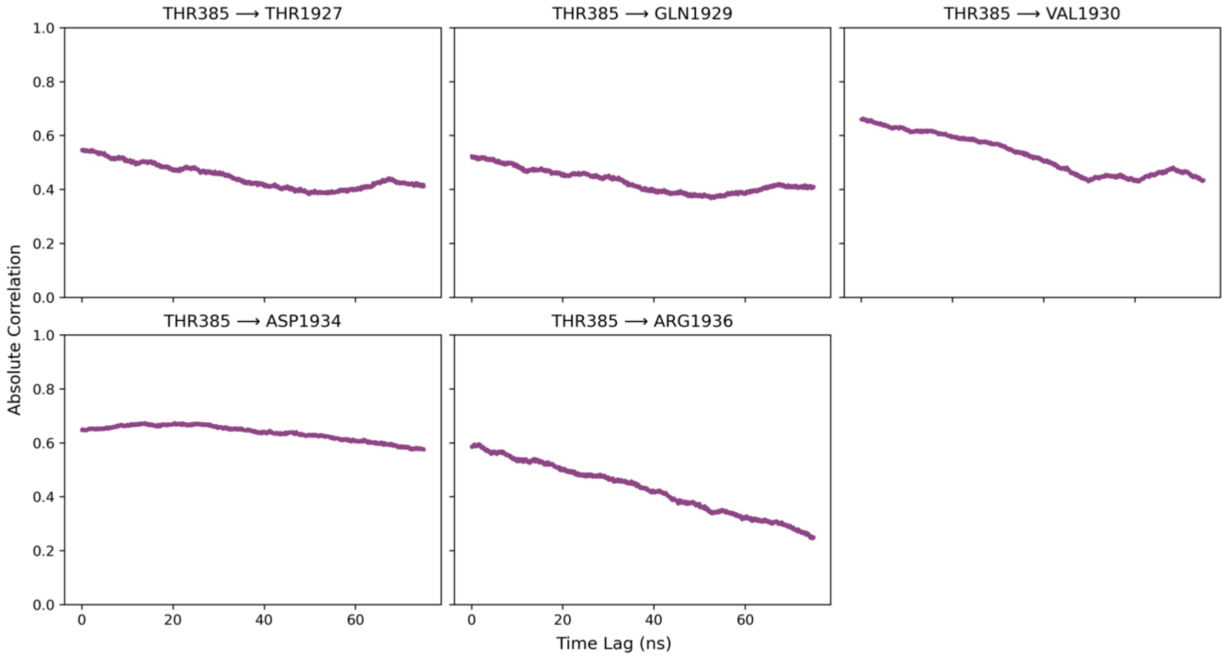
THR385 ⟶ THR1927
THR385 ⟶ GLN1929
THR385 ⟶ VAL1930
THR385 ⟶ ASP1934
THR385 ⟶ ARG1936
Absolute Correlation
Time Lag (ns)
0.0
0.2
0.4
0.6
0.8
1.0
0
20
40
60

G

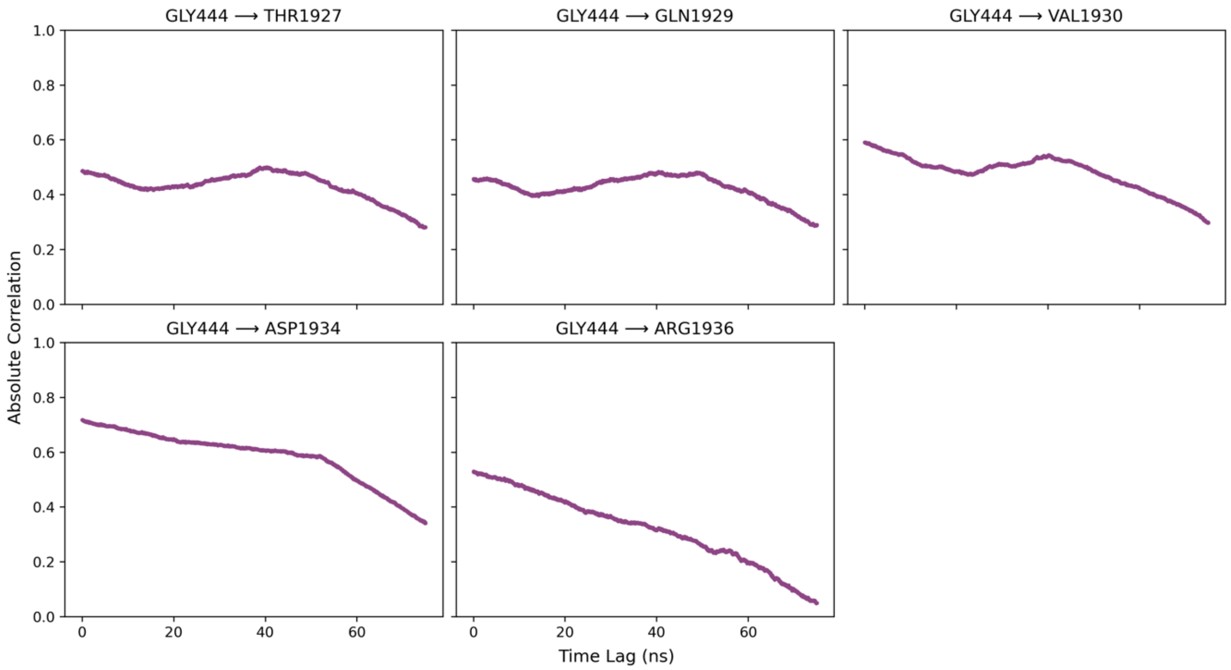

Supplemental Figure 9. Time-dependent torsional correlation plots for propagation pathways within the catalytic module, computed from the MD trajectory initialized with random seed 1. Arrows indicate the direction of propagation. **(A)** Arg9 correlations with CUL4A C-terminal residues. **(B)** Val1185 correlations with CUL4A C-terminal residues. **(C)** Asn1295 correlations with CUL4A C-terminal residues. **(D)** Ser1316 correlations with CUL4A C-terminal residues. **(E)** Ala384 correlations with RBX1 residues **(F)** Thr385 correlations with RBX1 residues. **(G)** Gly444 correlations with RBX1 residues.

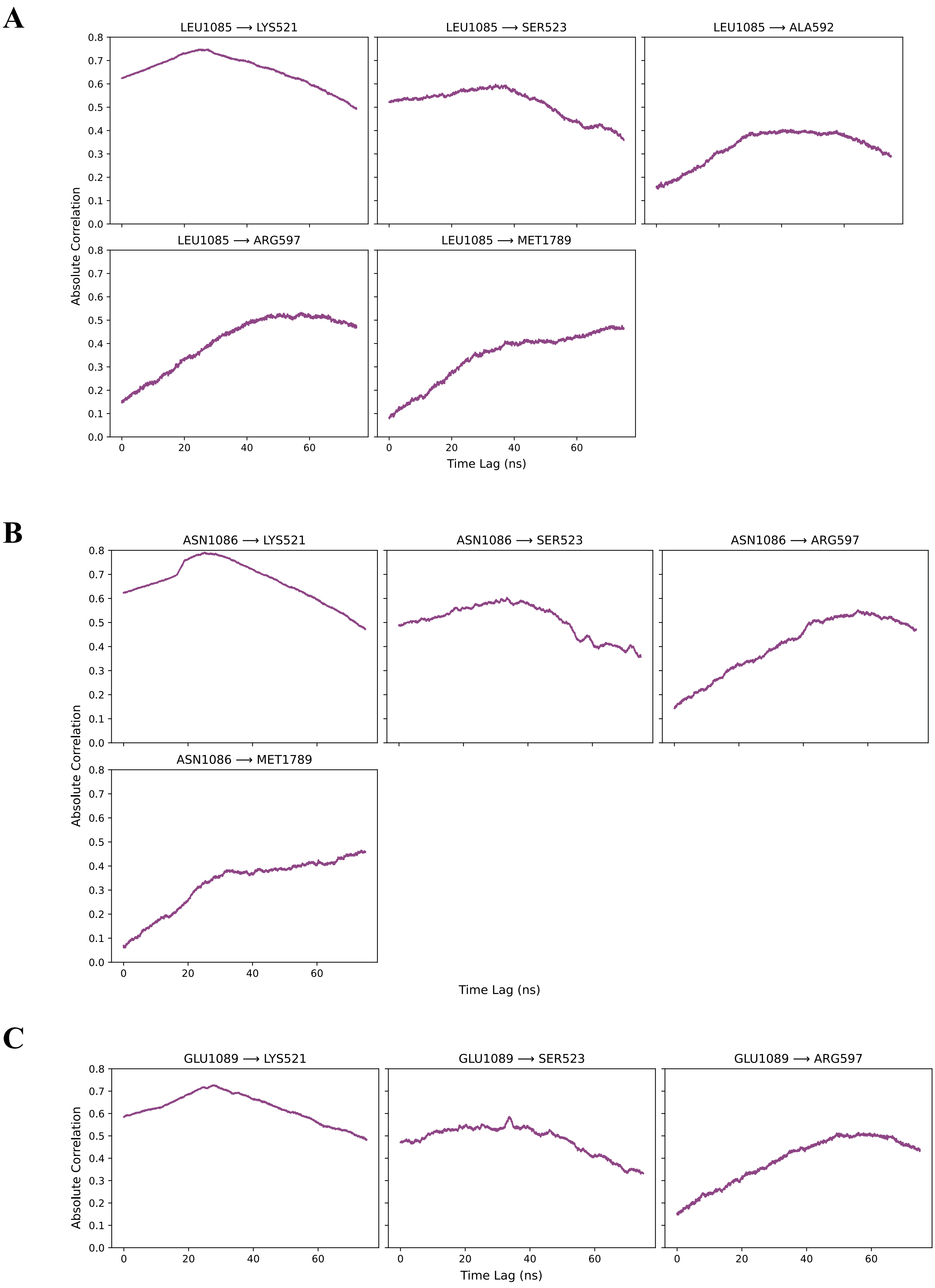
A
LEU1085 ⟶ LYS521
LEU1085 ⟶ SER523
LEU1085 ⟶ ALA592
LEU1085 ⟶ ARG597
LEU1085 ⟶ MET1789
Absolute Correlation
Time Lag (ns)
B
ASN1086 ⟶ LYS521
ASN1086 ⟶ SER523
ASN1086 ⟶ ARG597
ASN1086 ⟶ MET1789
Absolute Correlation
Time Lag (ns)
C
GLU1089 ⟶ LYS521
GLU1089 ⟶ SER523
GLU1089 ⟶ ARG597
Absolute Correlation
Time Lag (ns)

D

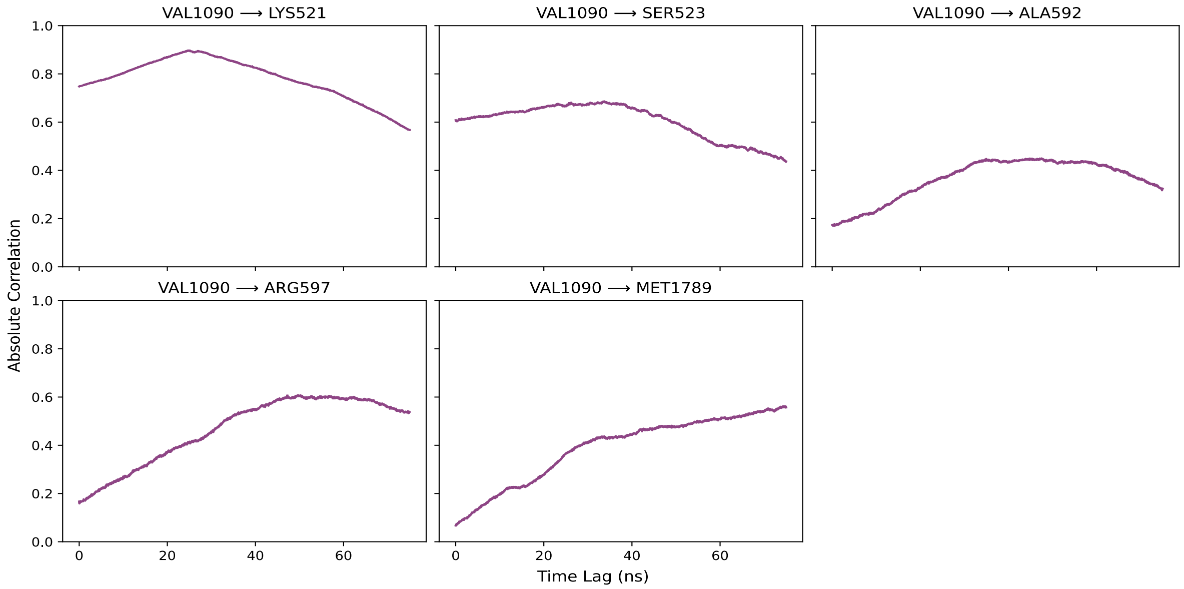
VAL1090 ⟶ LYS521
VAL1090 ⟶ SER523
VAL1090 ⟶ ALA592
VAL1090 ⟶ ARG597
VAL1090 ⟶ MET1789
Absolute Correlation
Time Lag (ns)

E

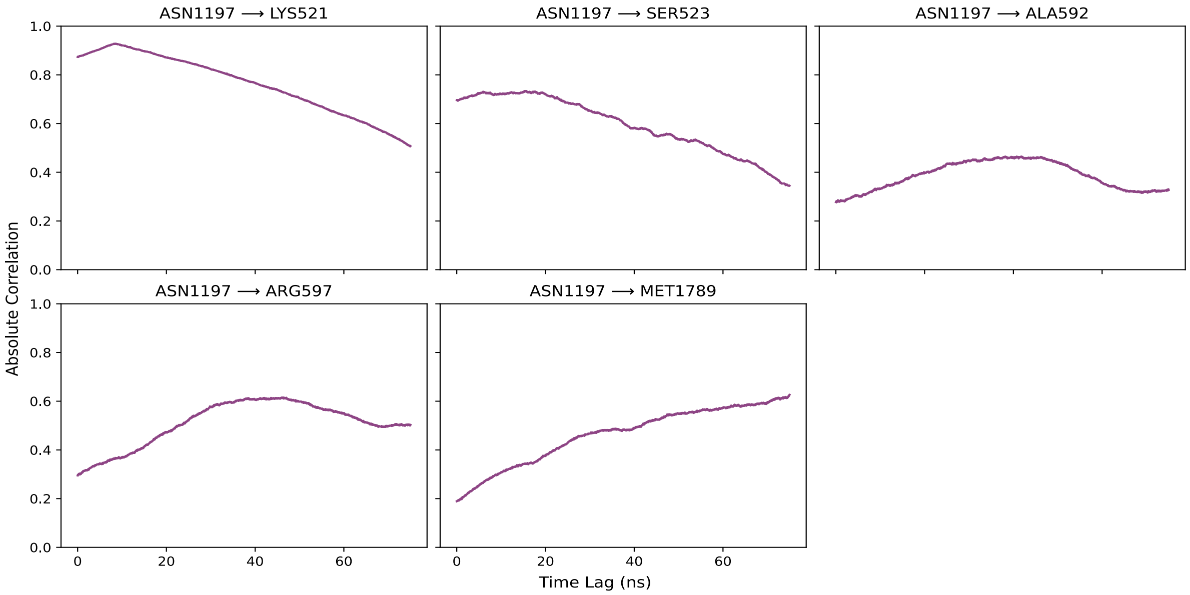
ASN1197 ⟶ LYS521
ASN1197 ⟶ SER523
ASN1197 ⟶ ALA592
ASN1197 ⟶ ARG597
ASN1197 ⟶ MET1789
Absolute Correlation
Time Lag (ns)

F

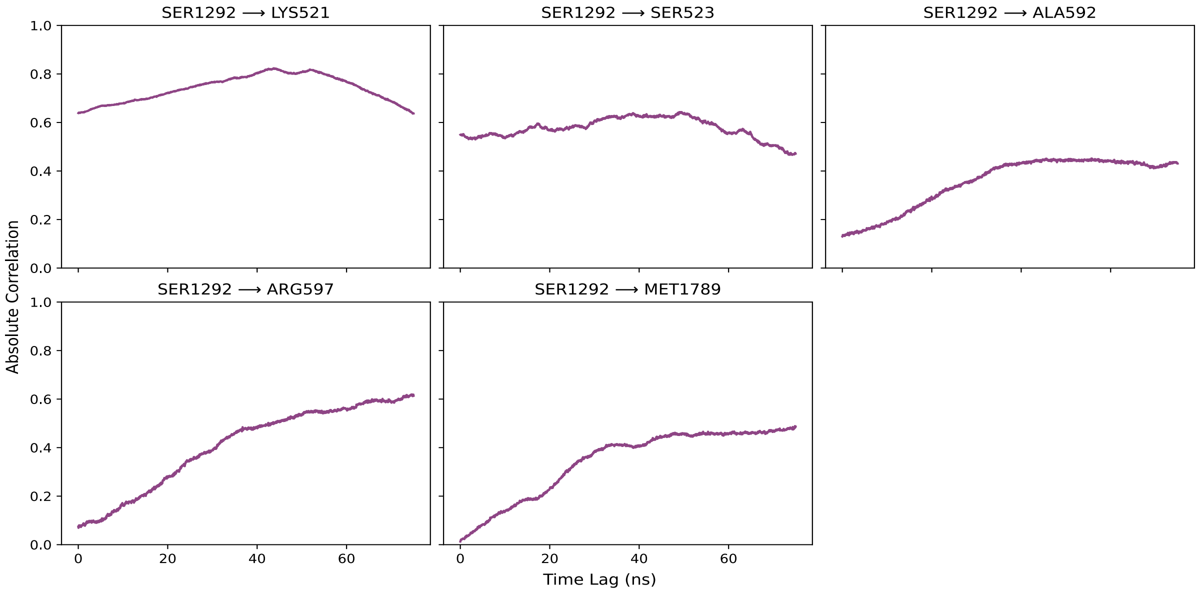
SER1292 ⟶ LYS521
SER1292 ⟶ SER523
SER1292 ⟶ ALA592
SER1292 ⟶ ARG597
SER1292 ⟶ MET1789
Absolute Correlation
Time Lag (ns)

G

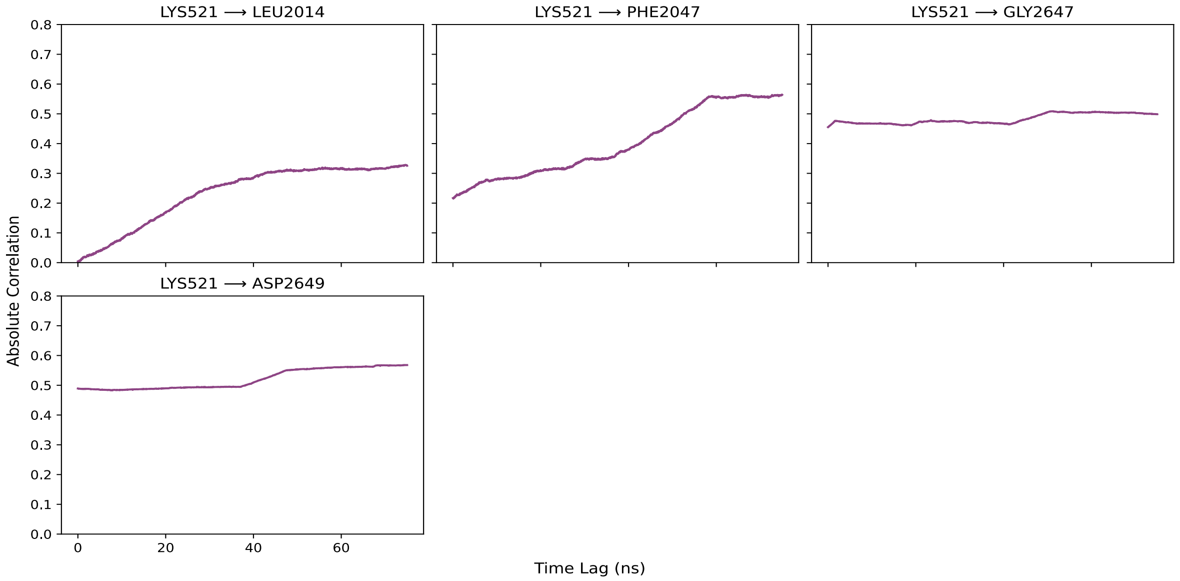

LYS521 ⟶ LEU2014
LYS521 ⟶ PHE2047
LYS521 ⟶ GLY2647
LYS521 ⟶ ASP2649
Absolute Correlation
Time Lag (ns)

H

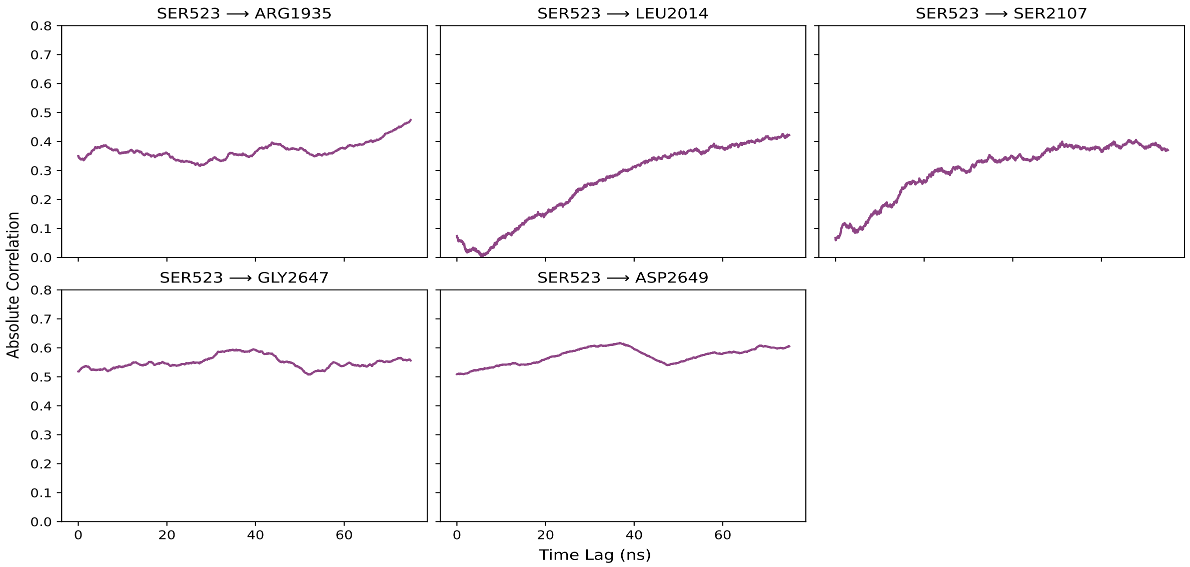

SER523 ⟶ ARG1935
SER523 ⟶ LEU2014
SER523 ⟶ SER2107
SER523 ⟶ GLY2647
SER523 ⟶ ASP2649
Absolute Correlation
Time Lag (ns)

I

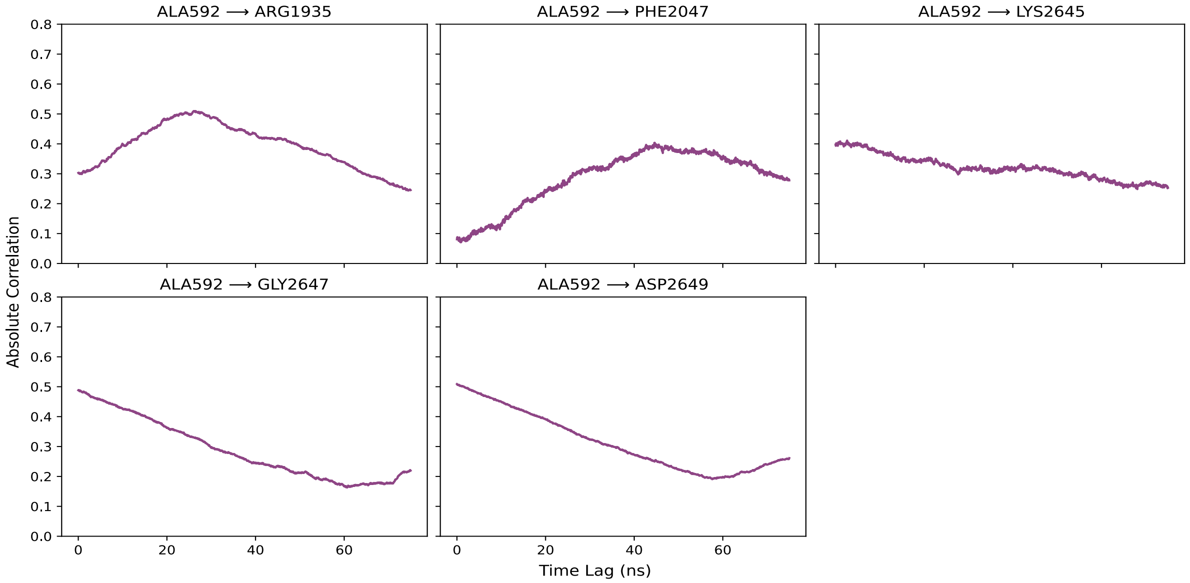

ALA592 ⟶ ARG1935
ALA592 ⟶ PHE2047
ALA592 ⟶ LYS2645
ALA592 ⟶ GLY2647
ALA592 ⟶ ASP2649
Absolute Correlation
Time Lag (ns)

J

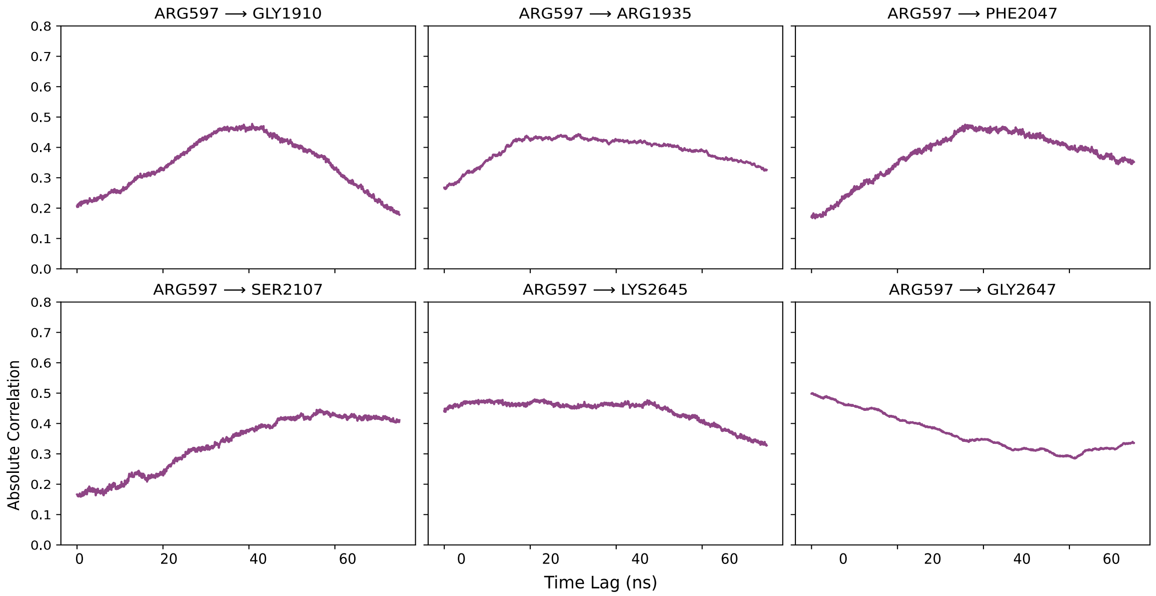

K

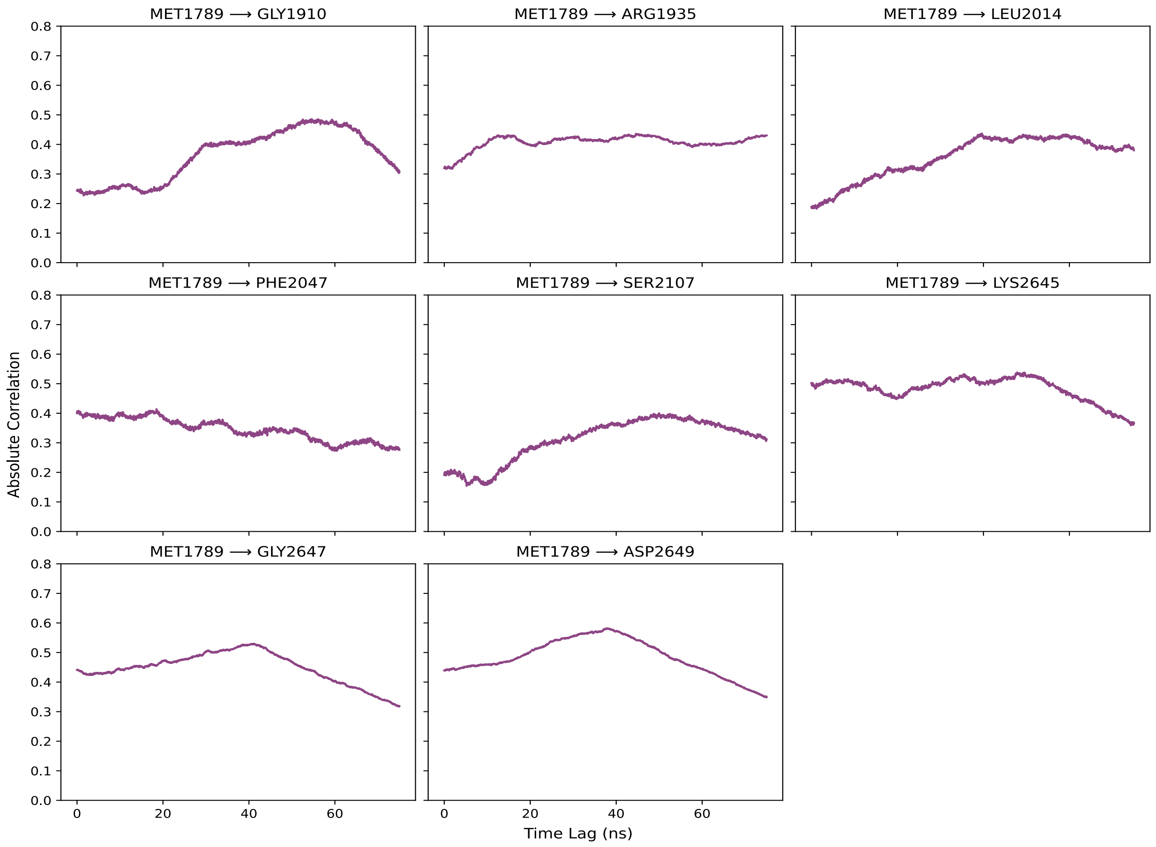

Supplemental Figure 10. Time-dependent torsional correlation plots for propagation pathways within the catalytic module, computed from the MD trajectory initialized with random seed 2. Arrows indicate direction of propagation. **(A)** Correlations for Leu1085 with residues within CUL4A C-terminal. **(B)** Asn1086 with residues within CUL4A C-terminal. **(C)** Glu1089 with residues within CUL4A C-terminal. **(D)** Val1090 with residues within CUL4A C-terminal. **(E)** Asn1197 with residues within CUL4A C-terminal**. (F)** Ser1292 with residues within CUL4A C-terminal. **(G)** Correlations for Lys521 with residues within the RBX1-UB-BRD4 interface. **(H)** Ser523 with residues within the RBX1-UB-BRD4 interface. **(I)** Ala592 with residues within the RBX1-UB-BRD4 interface. **(J)** Arg597 with residues within the RBX1-UB-BRD4 interface. **(K)** Met1789 with residues within the RBX1-UB-BRD4 interface.

A

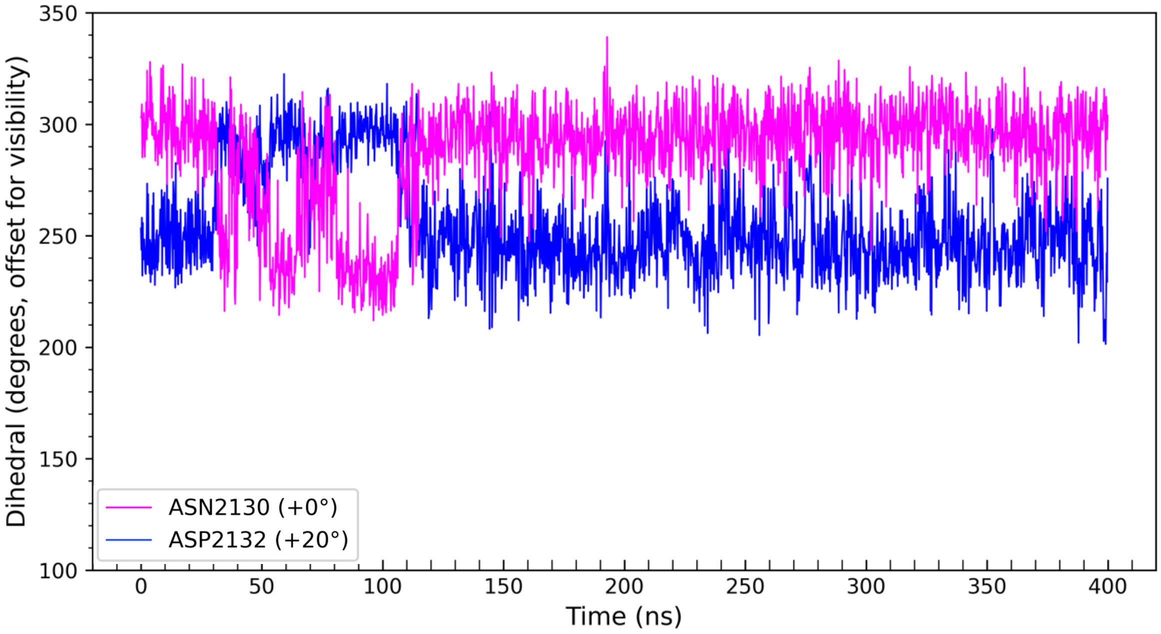

B

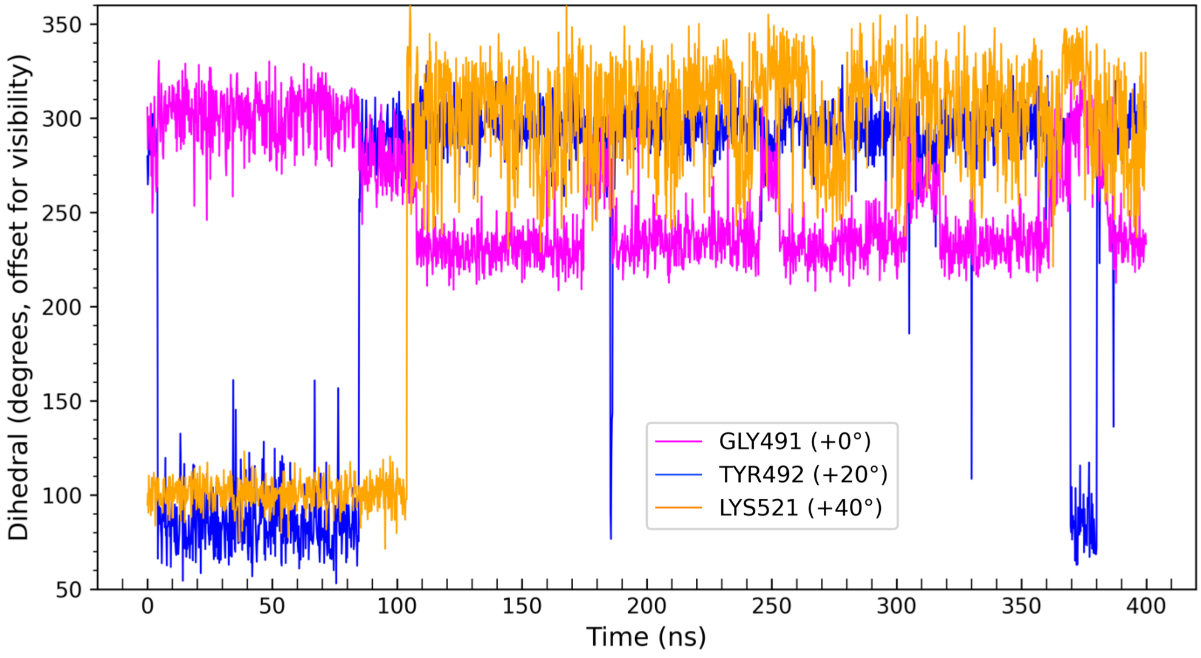

Supplemental Figure 11. Raw φ dihedral data for residues associated with the E2–BRD4 interface and the CUL4A C-terminal region, computed from the MD trajectory initialized with random seed 1. **(A)** Residues within the E2 interface with BRD4. **(B)** Residues within the CUL4A C-terminal region.

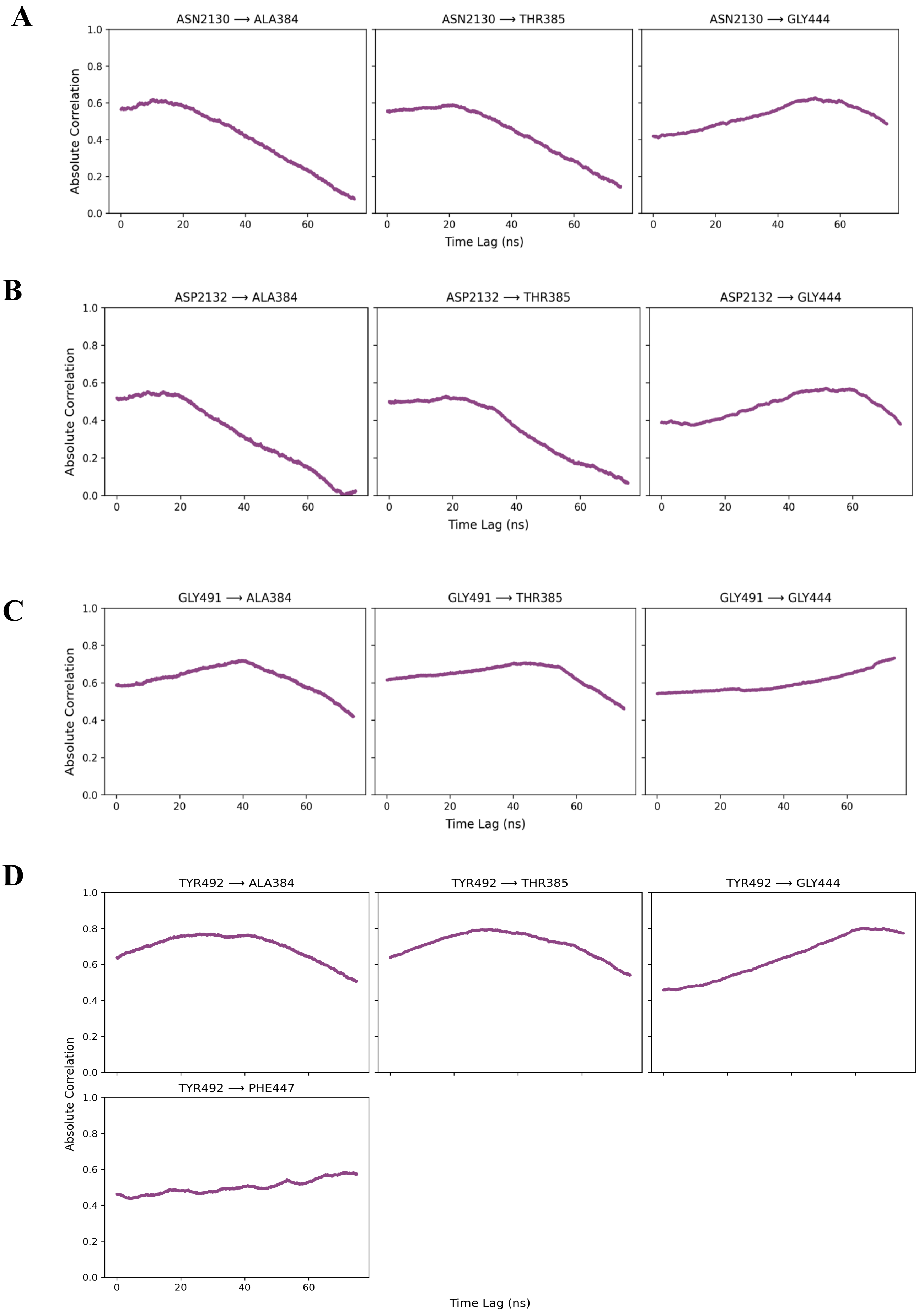
A
ASN2130 ⟶ ALA384
ASN2130 ⟶ THR385
ASN2130 ⟶ GLY444
Absolute Correlation
Time Lag (ns)
B
ASP2132 ⟶ ALA384
ASP2132 ⟶ THR385
ASP2132 ⟶ GLY444
Absolute Correlation
Time Lag (ns)
C
GLY491 ⟶ ALA384
GLY491 ⟶ THR385
GLY491 ⟶ GLY444
Absolute Correlation
Time Lag (ns)
D
TYR492 ⟶ ALA384
TYR492 ⟶ THR385
TYR492 ⟶ GLY444
TYR492 ⟶ PHE447
Absolute Correlation
Time Lag (ns)

E

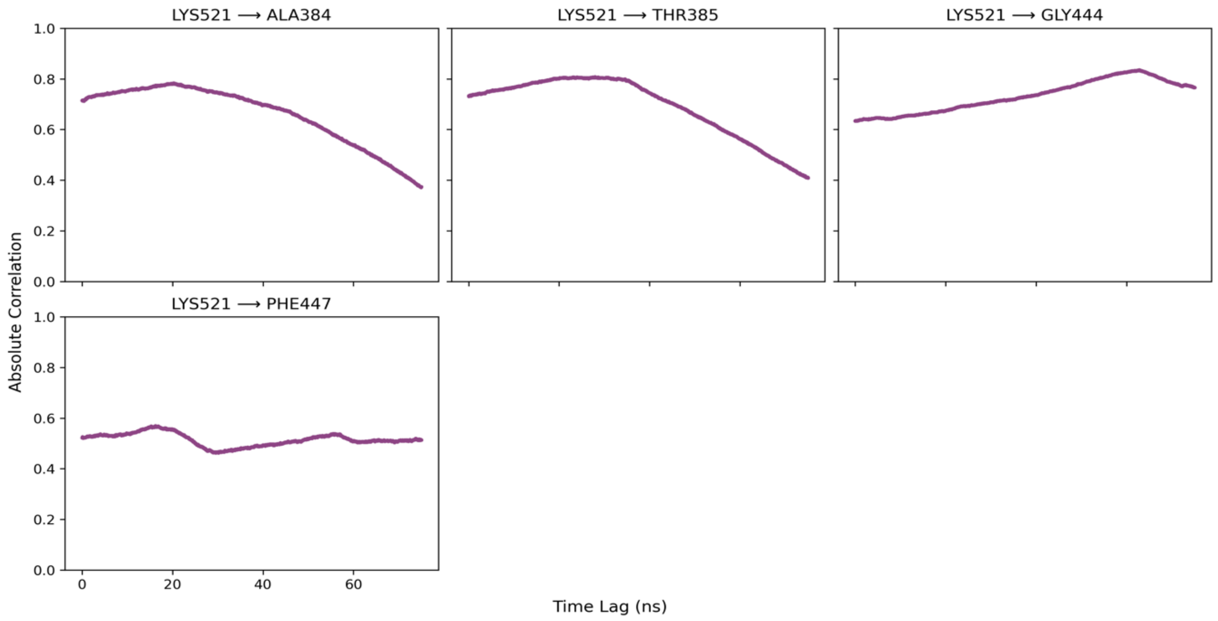

Supplemental Figure 12. Time-dependent torsional correlation plots for propagation pathways originating from additional regions of the catalytic module, computed from the MD trajectory initialized with random seed 1. Arrows indicate direction of propagation. **(A)** Correlations for Asn2130 with CUL4A C-terminal residues. **(B)** Asp2132 with CUL4A C-terminal residues. **(C)** Gly491 with CUL4A C-terminal residues. **(D)** Tyr492 with CUL4A C-terminal residues. **(E)** Lys521 with CUL4A C-terminal residues.

References

[1] Jenkins, G. M. and Watts, D. G. Spectral Analysis and Its Applications. Holden Day, 1968, print, ISBN-10: 0816244642.

[2] Tang, Z. and Chang, C. A. Systematic Dissociation Pathway Searches Guided by Principal Component Modes. J. Chem. Theory Comput., vol. 13, no. 5, pp. 2230-44, 2017, doi: 10.1021/acs.jctc.6b01204.

[3] Jenkins, G. M. and Watts, D. G. Spectral Analysis and Its Applications. Holden Day, 1968, print, ISBN-10: 0816244642.

[4] National Institute of Standards and Technology. Engineering and Statistics Handbook: Exploratory Data Analysis – Autocorrelation. Chapter 1.3.5.12.

[5] Lange, O. F. and Grubmüller, H. Generalized correlation for biomolecular dynamics. Proteins, vol. 62, no. 4, pp. 1053-1061, 2006, doi: 10.1002/prot.20784.

[6] Wu, K.Y.; Hung, T.I.; Chang, C.A. PROTAC-Induced Protein Structural Dynamics in Targeted Protein Degradation. eLife 13:RP101127, 2024, doi: 10.7554/eLife.101127.

[7] Maier, J. A.; Martinez, C.; Kasavajhala, K.; Wickstrom, L.; Hauser, K. E.; and Simmerling, C. ff14SB: improving the accuracy of protein side chain and backbone parameters from ff99SB. Journal of Chemical Theory and Computation, vol. 11, pp. 3696–3713, 2015, doi: 10.1021/acs.jctc.5b00255.

[8] He, X.; Man, V. H.; Yang, W.; Lee, T. S.; and Wang, J. A fast and high-- quality charge model for the next generation general AMBER force field. The Journal of Chemical Physics, vol. 153, 2020, doi: 10.1063/5.0019056.

[9] Jorgensen, W. L.; Chandrasekhar, J.; Madura, J. D.; Impey, R. W.; and Klein, M. L. Comparison of simple potential functions for simulating liquid water. The Journal of Chemical Physics, vol. 79, pp. 926–935, 1983, doi: 10.1063/1.445869.

[10] Essmann, U.; Perera, L.; Berkowitz, M. L.; Darden, T.; Lee, H.; and Pedersen, L. G. A smooth particle mesh Ewald method. The Journal of Chemical Physics, vol. 103, pp. 8577–8593, 1995, doi: 10.1063/1.470117.

[11] Ryckaert, J. P.; Ciccotti, G.; and Berendsen, H. J. C. Numerical integration of the cartesian equations of motion of a system with constraints: molecular dynamics of n-- alkanes. Journal of Computational Physics, vol. 23, pp. 327–341, 1977, doi: 10.1016/0021-9991(77)90098-5.

[12] Humphrey, W.; Dalke, A.; Schulten, K. VMD: Visual Molecular Dynamics. J. Mol. Graph, vol. 14, no. 1, pp. 33–38, 1996, doi: 10.1016/0263-7855(96)00018-5.

[13] Ai, R.; Fatmi, Q.; and Chang, C. A. T-Analyst: a program for efficient analysis of protein conformational changes by torsion angles. J. Comput. Aided Mol. Des., vol. 24, no. 10, pp. 819 27, 2010, doi: 10.1007/s10822-010-9376-y.

[14] Newman, M. E. J. Networks: An Introduction. Illustrated reprint, Oxford University Press, 2010. ISBN-10: 0191594172.

[15] Sethi, A.; Eargle, J.; Black, A. A; Luthey-Schulten, Z. Dynamical networks in tRNA:protein complexes, Proc. Natl. Acad. Sci. U.S.A., vol. 106, no. 16, pp. 6620-6625, 2009, doi: 10.1073/pnas.0810961106.

[16] Watts, D. J.; Strogatz, S. H. Collective Dynamics of “Small-World” Networks. Nature vol. 393, no. 6684, pp. 440–442, 1998, doi: 10.1038/30918.

[17] Ravasz, E.; Somera, A. L.; Mongru, D. A.; Oltvai, Z. N.; Barabási, A. L. Hierarchical Organization of Modularity in Metabolic Networks. Science, vol. 297, no. 5586, pp. 1551-1555, 2002, doi: 10.1126/science.1073374.

[18] Barabási, A. L.; Albert, R. Emergence of Scaling in Random Networks. Science, vol. 186, no. 5439, pp. 509-512, 1999, doi: 10.1126/science.286.5439.509.

[19] Newman, M. E. J. Modularity and Community Structure in Networks. Proc. Natl. Acad. Sci. U.S.A., vol. 103, no. 23, pp. 8577-8582, 2006, doi: 10.1073/pnas.0601602103.
